# Detection of Neanderthal Adaptively Introgressed Genetic Variants That Modulate Reporter Gene Expression in Human Immune Cells

**DOI:** 10.1093/molbev/msab304

**Published:** 2021-10-18

**Authors:** Evelyn Jagoda, James R Xue, Steven K Reilly, Michael Dannemann, Fernando Racimo, Emilia Huerta-Sanchez, Sriram Sankararaman, Janet Kelso, Luca Pagani, Pardis C Sabeti, Terence D Capellini

**Affiliations:** 1 Department of Human Evolutionary Biology, Harvard University, Cambridge, MA, USA; 2 Department of Organismic and Evolutionary Biology, Harvard University, Cambridge, MA, USA; 3 Broad Institute of MIT and Harvard, Cambridge, MA, USA; 4 Department of Evolutionary Genetics, Max Planck Institute for Evolutionary Anthropology, Leipzig, Germany; 5 Estonian Biocentre, Institute of Genomics, University of Tartu, Tartu, Estonia; 6 Lundbeck GeoGenetics Centre, The Globe Institute, University of Copenhagen, Copenhagen, Denmark; 7 Department of Ecology and Evolutionary Biology, Brown University, Providence, RI, USA; 8 Center for Computational Molecular Biology, Brown University, Providence, RI, USA; 9 Department of Computer Science, UCLA, Los Angeles, CA, USA; 10 Department of Human Genetics, UCLA, Los Angeles, CA, USA; 11 Department of Biology, University of Padova, Padova, Italy; 12 Department of Immunology and Infectious Diseases, Harvard T.H. Chan School of Public Health, Boston, MA, USA; 13 Howard Hughes Medical Institute, Chevy Chase, MD, USA

**Keywords:** neandertal, introgression, massively parallel reporter assay, positive selection, adaptation, immune

## Abstract

Although some variation introgressed from Neanderthals has undergone selective sweeps, little is known about its functional significance. We used a Massively Parallel Reporter Assay (MPRA) to assay 5,353 high-frequency introgressed variants for their ability to modulate the gene expression within 170 bp of endogenous sequence. We identified 2,548 variants in active putative *cis*-regulatory elements (CREs) and 292 expression-modulating variants (emVars). These emVars are predicted to alter the binding motifs of important immune transcription factors, are enriched for associations with neutrophil and white blood cell count, and are associated with the expression of genes that function in innate immune pathways including inflammatory response and antiviral defense. We combined the MPRA data with other data sets to identify strong candidates to be driver variants of positive selection including an emVar that may contribute to protection against severe COVID-19 response. We endogenously deleted two CREs containing expression-modulation variants linked to immune function, rs11624425 and rs80317430, identifying their primary genic targets as *ELMSAN1*, and *PAN2* and *STAT2*, respectively, three genes differentially expressed during influenza infection. Overall, we present the first database of experimentally identified expression-modulating Neanderthal-introgressed alleles contributing to potential immune response in modern humans.

## Introduction

As modern humans dispersed out of Africa, they interbred with Neanderthals, resulting in a contribution of Neanderthal DNA to the human genome pool that today comprises ∼2% of all non-African genomes (Prüfer et al. 2017). Evidence suggests that Neanderthal introgression was initially under purifying selection in human populations (Harris and Nielsen 2016; Juric et al. 2016) leading to a reduction in the Neanderthal genetic contribution over time (Fu et al. 2016) but that this purifying selection may have been constrained to only the first ten generations following interbreeding (Petr et al. 2019). Despite this, it has also been shown that some archaic hominin introgressed genetic variation experienced positive selection in modern human populations (Gittelman et al. 2016; Sankararaman et al. 2016; Racimo et al. 2017; Jagoda et al. 2018). Positive selection on introgressed variants occurred either soon after interbreeding with archaic humans and/or after modern humans expanded throughout Eurasia (Jagoda et al. 2018; Zhang et al. 2020; Yair et al. 2021). Given that Neanderthals lived in Eurasia 200,000 years before modern humans dispersed out of Africa, Neanderthals likely acquired genetic adaptations to Eurasian environmental stressors, including Eurasian-specific pathogens that modern humans living in Africa had never encountered. By breeding with these archaic hominins, modern humans may have acquired genetic variants that helped them adapt to these new environments. Notably, immunological pathways have often been highlighted as targets of selection on archaic variants. Archaic introgression is enriched in loci active in the immune system (Deschamps et al. 2016; Enard and Petrov 2018), particularly the innate immune system, as introgressed regions with clear evidence of positive selection are associated with differential expression of *Toll-Like Receptor* (Dannemann et al. 2016) and *OAS* antiviral genes (Sams et al. 2016), among others. Given the importance of the interaction between host genetics and immune response (Nedelec et al. 2016; Quach et al. 2016), functionally characterizing this introgressed component of variation may have important biomedical ramifications.

However, characterization of the functional consequences of introgressed genetic variation on the present-day human cells remains limited. Given that only a small portion of Neanderthal introgression affects protein coding sequences (Dannemann et al. 2017), a few studies have used computational approaches like expression quantitative trait loci (eQTL) (Dannemann et al. 2017), allele-specific expression (McCoy et al. 2017), and genome-wide association studies (GWAS) (Simonti et al. 2016; Dannemann and Kelso 2017) analyses to link introgressed variants to differential gene expression and organismal level phenotypes. However, because adaptive introgressed haplotypes are typically large (10s–100s of kb) and consist of many genetic variants that are inherited together (Racimo et al. 2017), it is difficult to know a priori which of the linked variants are the “causal” or “driver” variant(s) driving positive selection and which are nonfunctional hitchhikers on the selected haplotype (Nielsen et al. 2007). Without knowing the identity of the true causal variant(s) and importantly its functional effect(s), its phenotypic consequence and potential adaptive benefit cannot be fully understood. Here, we describe a genome-wide, high-throughput functional screen of noncoding genetic variants within putatively positively selected introgressed haplotypes present in at least one of 20 modern human populations from around the world. We directly assayed 5,353 variants for regulatory activity within K562 cells, a multipotent hematopoetic, and immune cell type, and identified 2,548 variants that fall within active *cis*-regulatory elements (CREs) and 292 variants that potentially modulate gene expression. This work represents the first high-throughput functional screen directly targeted at Neanderthal introgressed variants and reveals a set of directly identified putative causal variants, many of which display substantial additional evidence of affecting important immune pathways and being potential drivers of positive selection signals across the globe. Our results not only provide insights into the effects of adaptive archaic variation but are paramount toward a deeper understanding of the consequences of archaic introgression in modern humans.

## Results

### Variant Selection Scheme Prioritizes Putative Adaptively Introgressed Variants from a Worldwide Data Set

Since many putative adaptive introgressed variants show signs of local adaption (Huerta-Sánchez et al. 2014; Gittelman et al. 2016; Racimo et al. 2017; Jagoda et al. 2018), we aimed to maximize our ability to find functionally beneficial signals by incorporating variants from a diverse set of populations. We therefore created a variant selection scheme to incorporate data sets from the 1000 Genomes Project (1000 Genomes Project Consortium 2015), the Simons Diversity Project (Mallick et al. 2016), and the Estonian Biocentre Human Genome Diversity Panel (Pagani et al. 2016) to identify variants with a high likelihood of being both introgressed from Neanderthals and positively selected (see Materials and Methods). Furthermore, because this experimental assay uses synthetically created nucleotide sequences, we are not limited by sample collection as are large-scale genotype/phenotype databases (e.g., UK Biobank, Sudlow et al. 2015; GTEx, GTEx Consortium 2015), which primarily contain data from people with European ancestry. We therefore have a unique opportunity to identify functionally important variants from populations that have not been tested via computational methods alone. The sampled populations include representation from Europe, East Asia, South Asia, the Americas, and Melanesia (supplementary table S1, Supplementary Material online).

We prioritized variants previously identified as introgressed using the Altai Neanderthal genome (Sankararaman et al. 2016; Vernot et al. 2016; Racimo et al. 2017; Jagoda et al. 2018) that have elevated frequency, defined as ≥20% in at least one of the 20 populations studied. From this pool of 59,955 variants, we examined a reduced (∼10%), randomly selected, variant set to test via a Massively Parallel Reporter Assay (MPRA) (Tewhey et al. 2016). We prioritized the 1,156 that had been previously determined to be introgressed eQTLs in relevant tissue types (Dannemann et al. 2017) and selected an additional 4,197 variants randomly from the remaining pool, leading to a total of 5,353 variants. These variants span 2,846 unique 40 kb windows (the average size of a confidently introgressed haplotype of the genome; Huerta-Sánchez et al. 2014). Although the majority of these variants fall in intergenic regions, those variants that fall within K562 Ensembl genomic annotations (Hunt et al. 2018) are shown in (supplementary fig. 1, Supplementary Material online). Here, most of these annotated variants fall in promoter flanking regions (12%), followed by CTCF-binding cites (3%), enhancers and promoters (2% each), open chromatin regions (1%), and transcription factor (TF)-binding sites (0.3%). Overall, this variant set represents the most geographically diverse set of putatively positively selected introgressed variants used in a single functional analysis of Neanderthal introgression. We note that by focusing on introgressed variants for expression modulation, we may be missing important functional contributions from linked human derived alleles.

### MPRA Design and Implementation

**Fig. 1.**
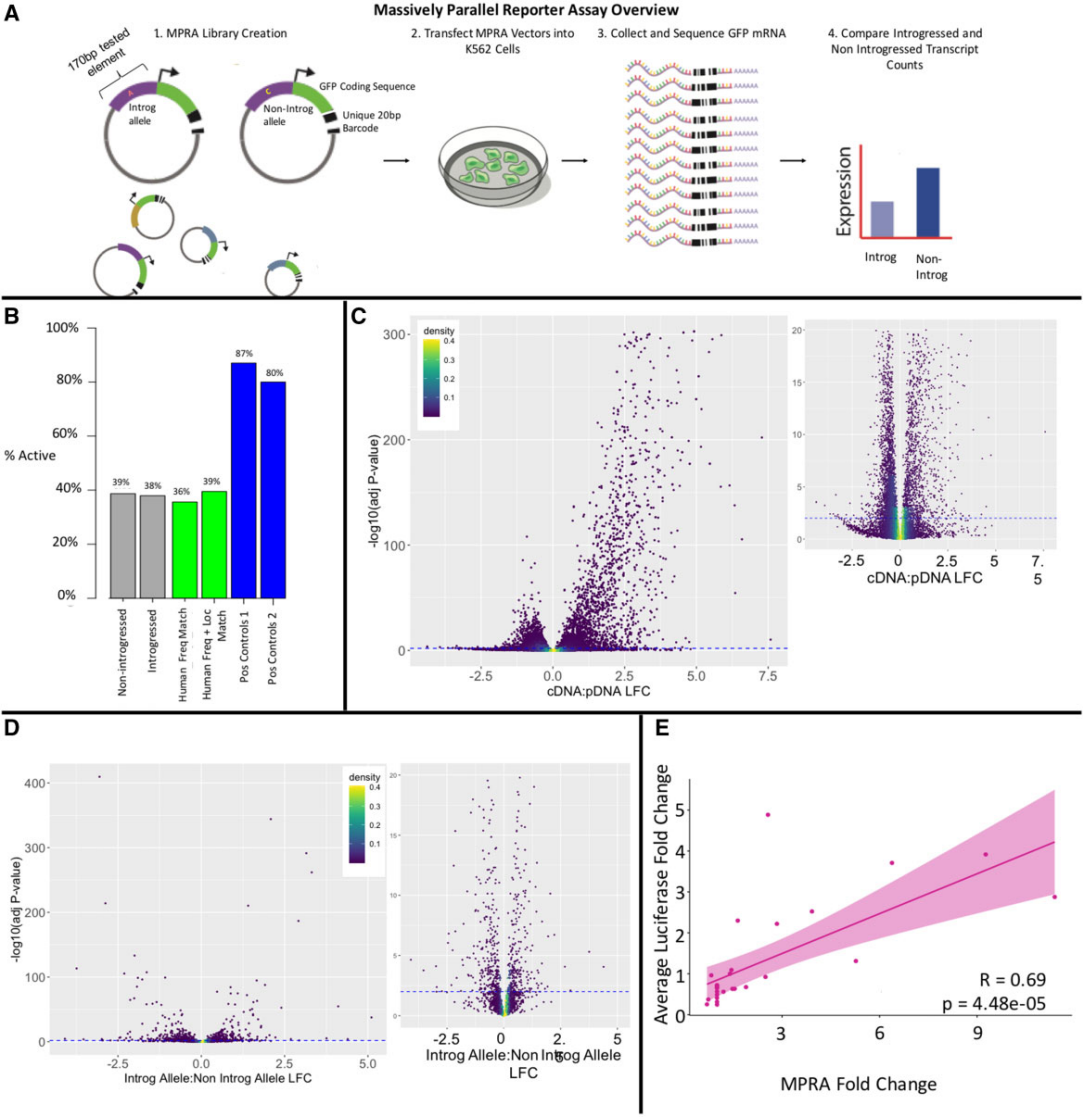
Massively parallel reporter assay overview and results. (*A*) Experimental overview. Following Tewhey et al. (2016), MPRA was conducted through the creation of reporter vectors containing either the introgressed or nonintrogressed allele at a locus along with 85 bp of endogenous flanking sequence on either side including any variants linked at *r*_2_>0.9 to the variant of interest. These experimental oligo sequences are fixed with unique 20 bp barcode sequences and cloned into reporter vectors containing the GFP coding sequence and a minimal promoter. The whole vector pool is transfected into K562 cells and resultant GFP mRNA is collected and sequenced. Transcripts are linked to the experimental sequence via the transcribed barcode and normalized introgressed and nonintrogressed transcript counts are compared. (*B*) MPRA activity results. Number of active putative CREs across tested categories as defined in text. Pos Control 1 are active sequences from a prior MPRA in LCL cells (Ulirsch et al. 2016) and Pos Control 2 are active sequences from a prior MPRA in K562 cells (Sloan et al. 2016). (*C*) Volcano plots showing the distribution of activity for all tested elements sequences by comparing the cDNA:pDNA ratio LFC and calculating the adjusted *P* value. The blue dotted line represents the *P* value threshold −log10(0.01). Points about the line are considered significantly active CREs. The plot to the right is a zoomed-in version, showing just sequences with −log10(*P*)<20. (*D*) Volcano plots showing the distribution of expression modulation for variants in active CREs determined by comparing the introgressed to nonintrogressed allelic ratio LFC and calculating the adjusted *P* value. Points above the line are considered significantly emVars. The plot to the right is a zoomed-in version, showing just sequences with −log10(*P*)<20. (*E*) SLRA luciferase validation. *X* axis—activity results from MPRA reported as fold change RNA:DNA. *Y* axis—average luciferase activity normalized to empty luciferase vector across replicates. Pearson’s *r* = 0.6922, *P* = 4.479e-05.

We employed a MPRA technique following Tewhey et al. (2016) (fig. 1*A*) to test the regulatory effects of 5,353 introgressed alleles (hereafter, the introgressed set) and their corresponding 5,353 nonintrogressed allelic counterparts (hereafter, the nonintrogressed set). This approach assists in identifying putatively functional driver variants. Each allele was tested within a 170-bp sequence centered on the variant, with the full 170-bp sequence referred to as the “tested element.” Additionally, we included 301 positive control sequences that were shown to have regulatory activity via published MPRA experiments in the same or similar cell lines (LCL, Tewhey et al. 2016; K562, Ulirsch et al. 2016; see Materials and Methods). Finally, we included two human allele control sets to assess any uniqueness in the activity of loci containing introgressed alleles. One set, the “human frequency matched set” consisted of 233 human alleles with the same frequency distribution as the introgressed set. The other set, the “human frequency and location matched set” consisted of 552 human alleles with the same frequency distribution as the introgressed set and with each variant located between 200 and 1,000 bp of an experimental variant.

We next tested these 11,792 total sequences in an MPRA following Tewhey et al. (2016) (see Materials and Methods) using K562 cells. This K562 line was primarily chosen and focused upon here because its DNase I hypersensitivity site map (DHS) shows the highest number of overlaps with the introgressed variants among 208 tissues/cell lines with ENCODE DHS maps (Sloan et al. 2016) (supplementary fig. 2, Supplementary Material online), with 469 introgressed alleles falling within K562 DHS peaks. The K562 cell line was derived from chronic myelogenous leukemia and has been demonstrated to display properties of a multipotent hematopoietic cell, displaying lymphoblastic morphology and differentiating into several immune cell types (Butler and Hirano 2014; Simonti et al. 2016) including granulocytes and monocytes and has been shown to express immunological factors (Tabilio et al. 1983) along with displaying some erythrocyte properties (Ulirsch et al. 2016). Because the specific immune phenotypes driven by introgressed variation is not yet known, there is value in using a multipotent cell type which has the potential to possess a transcriptional environment relevant to a larger number of variants. This cell type is also easy to grow and transfect, making it ideal for this assay which demands a large number of transfected cells. Furthermore, the fact that there exist a multitude of data sets for this cell type, including ChIP-seq chromatin data, DNAse hypersensitivity data, and more allow for more accurate functional interpretation of MPRA results conducted on this data set. However, although this is a screen for assessing potential functional variants, many false negatives will be due to the failure to detect regulatory variants in this specific cell type.

For each of four biological replicates, the MPRA vector pool was transfected into K562 cells, and a normalized number of transcripts driven by each tested element was determined (see Materials and Methods). This transcript count was compared with the tested element’s representation in the DNA vector pool. If the log2-fold change (LFC) of this ratio significantly differed from 0, with an adjusted *P* value of less than 0.01, the element is considered “active” (Tewhey et al. 2016), in that it drove a different amount of transcription than would be expected by its representation in the DNA vector pool alone. These active sequences are referred to as active CREs. A full set of all tested sequences is shown in supplementary table S2, Supplementary Material online. The normalized cDNA counts for each tested element were highly reproducible across all experiments (*r* > 0.99, *P* < 2.2×10^−16^) (supplementary fig. 3, Supplementary Material online) as were the ratios of cDNA:pDNA across replicates (minimum *r* = 0.96, *P* < 2.2×10^−16^) (supplementary fig. 4, Supplementary Material online). Furthermore, nearly all of the positive control oligos, which had been shown to be active in MPRA assays in related cell lines, showed activity (87% and 80% for the two sets, fig. 1*B*). Moreover, the relative expression of the positive control oligos in their original assay is significantly correlated to their expression in our assay (positive controls originally tested in K562 cells, Ulirsch et al. 2016: Pearson’s *r* = 0.7, *P* = 2.5×10^−14^; in LCL cells, Tewhey et al. 2016: Pearson’s *r* = 0.47, *P* = 4.9×10^−13^, supplementary fig. 5*A* and *B*, Supplementary Material online). These findings suggest that the assay is both accurate and reproducible. Finally, analysis of the relationship between barcode count and coefficient of variation across experiments provided validation for the activity expression values (supplementary fig. 6, Supplementary Material online; see Materials and Methods).

### Nearly Half of Tested Elements Show Activity in K562 Cells

**Fig. 2.**
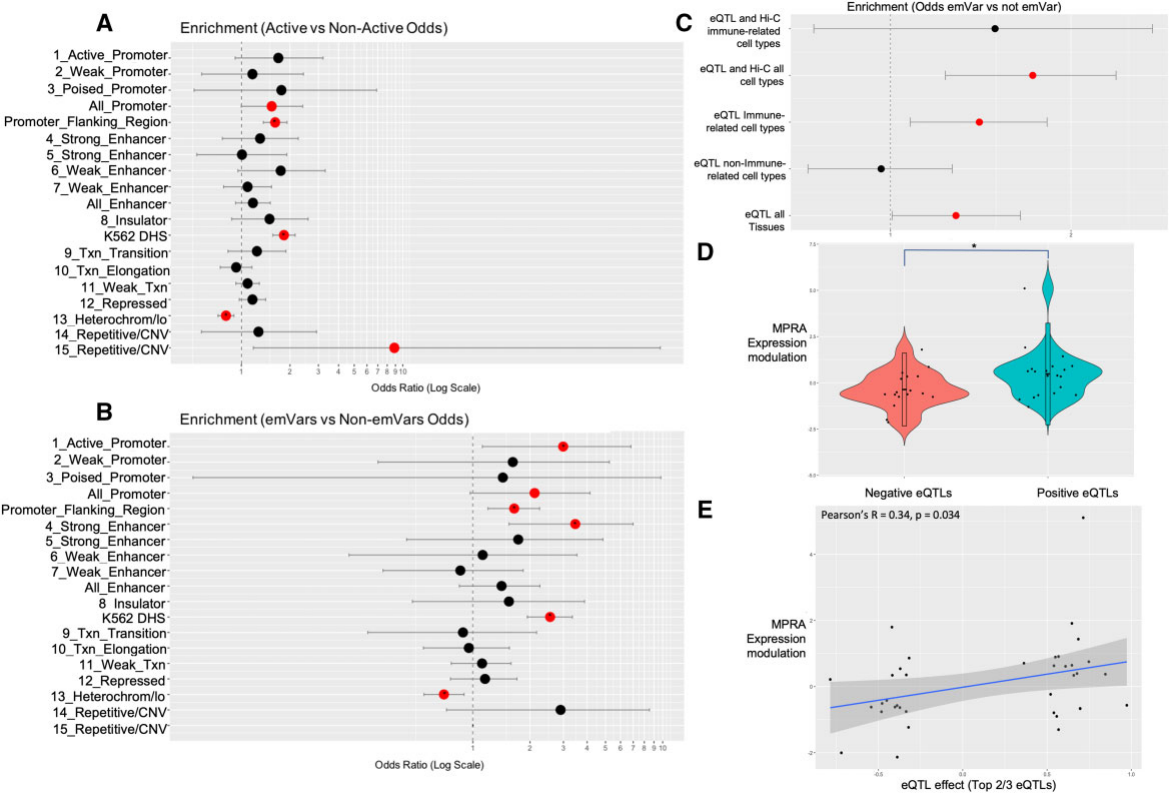
Active CREs and emVars display prosperities of endogenous K562 biology. (*A*) Enrichment of active CREs in K562 genomic features relative to nonactive sequences. (*B*) Enrichment of emVar sequences in K562 genomic features relative to non-emVar sequences. (*C*) Enrichment of emVars acting as eQTLs and eQTLs in the presence of evidence that they reside in a regulatory element that interacts with a target gene via Hi-C chromatin interaction data in immunological or any cell type. (*A*–*C*) Enrichments are reported as Fisher’s OR with lines indicating confidence intervals. Significant enrichments (*P* < 0.05) are colored in red. (*D*) Violin plots of MPRA expression modulation for emVars acting as downregulatory (negative) or upregulatory (positive) eQTLs (top two-third of eQTL signals). Positively associating eQTLs show significantly higher MPRA skew values than negatively associated eQTLs (*t*-test, *P* = 0.038). (*E*) Correlation between eQTL effect (top two-third of eQTLs) and MPRA expression modulation. We note there is an overall significant correlation between the eQTL effect and MPRA skew for this set (Pearson’s *r* = 0.34, *P* = 0.034).

The MPRA discovered that 2,548 (48%) of tested elements are in active CREs in either the introgressed and/or nonintrogressed form (supplementary table S3, Supplementary Material online). Tested elements with introgressed alleles had a similar chance of being active as tested elements with the corresponding nonintrogressed allele (38% vs. 39%) (fig. 1*B*). We find correspondence between the distribution of these active CREs and other K562 regulatory information, which suggests that MPRA-discovered active CREs are biologically relevant despite being identified in an episomal context. For example, active CREs are significantly more likely than nonactive tested elements to fall within K562 DHS (Sloan et al. 2016) (Fisher’s odds ratio [OR] = 1.8, *P* < 0.0001, FDR < 0.05) and promoter flanking regions (Hunt et al. 2018) (OR = 1.6, *P* < 0.0001, FDR < 0.05) (fig. 2*A*). These features of the genome reflect consistent *cis*-regulatory activity in K562 cells (Ernst and Kellis 2017). Furthermore, active MPRA CREs are also significantly depleted of heterochromatic marks (OR = 0.8, *P* < 0.0001, FDR < 0.05), which are indicative of nonactive genomic region (Ernst and Kellis 2017). Also consistent with K562 endogenous biology, MPRA CREs within regions of active promoter chromatin marks drive significantly higher levels of reporter gene expression than CREs in almost any other chromatin state (Student’s *t*-test *P* < 0.0004, FDR < 0.05) (supplementary fig. 7*A*, Supplementary Material online). Finally, chromatin capture data (Jung et al. 2019) shows that active regions are more likely than nonactive regions (Fisher’s OR = 1.2, *P* = 0.004) to interact with a promoter in one of three immune tissues (lymphoblasts, spleen, and thymus). These results suggest that the MPRA CREs display properties consistent with activity in the endogenous K562 context and therefore can provide insights into immune biology.

Both human variant control sets showed similar amounts of active CREs in the MPRA (frequency matched only: 35.6%; frequency matched and location matched: 39.5%) as the introgressed (37.8%) and nonintrogressed (38.6%) CRE sets (fig. 1*B*). This similar proportion of activity is consistent with the endogenous context of these human CRE sets which display similar levels of K562 DHS (Sloan et al. 2016) overlap as do the introgressed and nonintrogressed variant sets (supplementary fig. 8, Supplementary Material online). Moreover, there is also a strong, significant correlation (Pearson’s *r* = 0.97, *P* = 0.03) across these four sets between the percent DHS overlap and percent MPRA activity (supplementary fig. 8, Supplementary Material online). Overall, these result show that active CREs that have tolerated introgressed alleles in some populations do not deviate from their expected activity levels in K562 cells relative to CREs in their immediate vicinity within the regulatory landscape of K562 cells, and to that of CREs containing human-derived frequency matched alleles.

### 292 Introgressed Variants Modulated Reporter Gene Expression

Following confirmation that active CREs in the MPRA reflect K562 biology, we next identified variants within active CREs that significantly modulate gene expression. We defined these variants as those for which the difference between the activity of introgressed variant and the activity of the nonintrogressed orthologous variant was significant with an adjusted *P* value less than 0.01 (see Materials and Methods). We refer to such variants as “expression-modulating variants” (emVars) and we identified a total of 292 (or 5.5% of the total variant set; 11.5% of those within active CREs, supplementary table S4, Supplementary Material online). These 292 emVars spanned 170 unique 40 kb windows. In general, emVars showed no overall significant direction of effect based on introgression status and were equally likely to increase or decrease reporter expression in K562 cells (one-sample Wilcoxon test compared with 0, *P* = 0.4) (fig. 1*D* and supplementary fig. 9, Supplementary Material online). This is consistent with previous work that showed no significant direction of effect for introgressed alleles in any tissue type aside from brain and testes in which they were downregulated (McCoy et al. 2017). As support for the accuracy of the assay, both sets of positive control sequences showed significant correlations between the expression modulation in their original assay and here (Pearson’s *r* = 0.57, *P* = 0.0053; *r* = 0.68, *P* = 4.8×10^−6,^ respectively, supplementary fig. 5*C* and *D*, Supplementary Material online).

We found further support for the emVar data by conducting single-locus reporter assays (SLRAs). We conducted SLRAs on 14 tested sequences (0.26% of tested variants), exceeding the proportion tested in similar experiments (e.g., Tewhey et al. 2016 tested 0.039% of variants). We found a strong and significant correlation between the SLRA results and the MPRA results (Pearson’s *r* = 0.69, *P* = 4.48×10^−5^, fig. 1*E* and table 2). These data are an important validation of the MPRA results because they provide support for the determination of expression modulation in our data via an independent assay that measures the effects of the alleles on the magnitude of fluorescence of the luciferase, a proxy for protein abundance. The overall concordance suggests that the MPRA is generally accurate and that the differential transcript levels largely correlated to changes in protein abundance.

**Table 2.** Variants Tested in Both MPRA and Single-Locus Reporter Luciferase Assay.

SNP	rs	MPRA LFC^a^	SLRA LFC	eQTL for Immune Gene	Highest Frequency Pop (Data Set)
4:177148043	rs13129964	1.11	0.80***	*ASB5*	25% Southwest Europe (EGDP)
22:32895077	rs73170400	−0.40	−0.32**	*FBXO7*	25% Southwest Europe (EGDP)
11:112021767	rs5744258	0.94	0.97***	*IL18*	22% Europe (1KG)
12:56638077 12:56660905 12:56727705	rs74673257 rs60542959 rs80317430	0.81 −0.54 −0.65	NS −0.046 −0.76***	*IL23A, STAT2, PAN2*	50% Oceania (EGDP)
14:74134717 14:74123986 14:74209097 14:74109421 14:74110882 14:74134485 14:74152316 14:74204686	rs4635279 rs62004913 rs11624425 rs62004909 rs2007722 rs7152308 rs12436322 rs72721770	0.97 −0.30 −0.46 NS NS NS NS NS	NS NS −0.20 (*P* = 0.07) NS NS −0.37* NS NS	*PNMA1, ELMSAN1*	31% Americas (SGDP)

Note.—NS, not significant.

a All values represent significant expression modulation LFC in the MRPA.

* *P* < 0.05, ** *P* < 0.01, ***P < 0.001.

### emVars Show Concordance with Other Measures of Functionality

As with the active CREs, CREs with emVars display properties consistent with K562 biology. For example, CREs with emVars are more likely than elements without emVars to fall within promoter flanking regions (OR = 1.65, *P* < 0.01, FDR < 0.05) and K562 DHS sites (OR = 2.32, *P* < 0.001, FDR < 0.05) (fig. 2*C*). They were also more likely to display chromatin marks consistent with active promoters (OR = 2.99, *P* < 0.05, FDR < 0.05) and strong enhancers (OR = 3.46, *P* < 0.01, FDR < 0.05), these latter two enrichments were not found within just active CREs. CREs with emVars were also depleted for heterochromatin chromatin marks (OR = 0.7, *P* < 0.01). In addition, when considering the magnitude of expression modulation across genomic features, emVars within CREs that have chromatin marks consistent with strong enhancers modulated expression more strongly than those within CREs with weak enhancer chromatin marks, though this comparison did not pass multiple hypothesis testing correction (average absolute value expression modulation LFC = 1.17 and 0.48, respectively, Student’s *t*-test *P* value = 0.037) (supplementary fig. 7*B*, Supplementary Material online).

The emVar results also show consistency with eQTL analyses. Of the variants tested, 1,365 were GTEx-annotated eQTLs in the three most relevant tissues to K562 cells (whole blood, lymphocytes, spleen). About 93 of these variants were emVars, which marks a significant enrichment for being emVars (OR = 1.39, *P* = 0.013) (fig. 2*C*). Variants that are eQTLs in any tissue are also enriched for being emVars, but to a lesser degree (OR = 1.29, *P* = 0.041), and variants that are eQTLs in any tissue besides the three most relevant are not enriched for being emVars (*P* = 0.84) (fig. 2*C*), as expected given the cell type-specific nature of this experiment. Furthermore, emVars are even further enriched for being eQTLs for a gene for which they are also shown to interact via Hi-C in any tissue (OR = 1.73, *P* = 0.0014) (fig. 2*C*), although this enrichment is not significant in immune-related tissues specifically, likely due to the low absolute number of variants in this set. These emVars that show shared eQTL and Hi-C signals are strong functional candidates and are reported in supplementary tables S5 and S6, Supplementary Material online.

When considering only emVars that show the same direction of effect for any response gene in the three relevant tissue set, we find that 61% show the same direction of effect in the MPRA. Tewhey et al. (2016) found that concordance between MPRA and eQTL results is strengthened when the weakest one-third of eQTL results are removed. Similarly, we see a modest increase to 65% concordant direction in this set. Furthermore, in this set, introgressed eQTLs that are associated with upregulation of a gene show significantly more upregulation in the MPRA values than introgressed eQTLs associated with downregulation of a gene (expression modulation LFC mean for the upregulatory set = 0.48, for the downregulatory set = −0.36, *t*-test, *P* = 0.038) (fig. 2*D*). Finally, there is an overall significant correlation between the eQTL effect and MPRA skew for this set (Pearson’s *r* = 0.34, *P* = 0.034) (fig. 2*E*). We expect this to be an underestimate of the accuracy of our results, given that the eQTL analyses are conducted on a European data set and the geographic diversity of our variant pool.

**Fig. 3.**
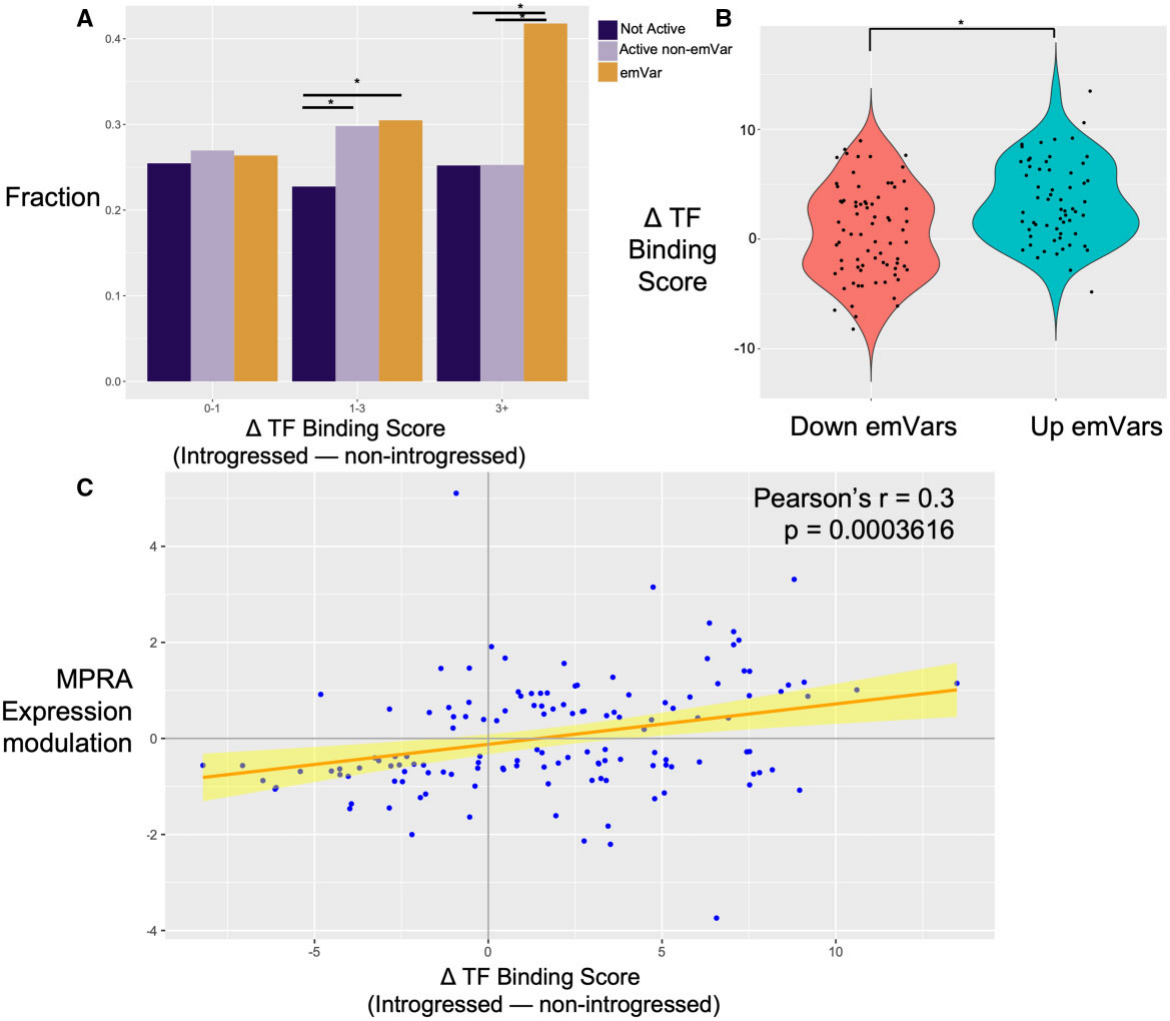
Transcription factor-binding site analysis of emVars. (*A*) After curating a set of predicted TF-binding motifs in K562 cells (see Materials and Methods) and examining at each introgressed and nonintrogressed sequence how each variant alters binding strength (deltaTF Binding score), we compared different classes of variants (not-active variants, active non-emVars, and emVars) for their overall impacts on differential binding. In general, emVars are more likely to lead to both large changes (3+) in predicted binding strength than either nonactive sequences (OR=1.91, *P* = 2.1×10^−5^) or active sequences with no emVar (OR=1.93, *P* = 2.9×10^−5^). emVars and active sequences with no emVar are both more likely than nonactive sequences to have moderate changes (1–3) in predicted binding strength (OR emVar vs. nonactive=1.42, *P* = 2.4×10^−5^, OR active vs. nonactive=1.36, *P* = 0.002). (*B*) We then compared binding affinities at each variant in each emVar to their direction of effect on expression in MPRA. In general, emVars that upregulated expression in the MPRA were predicted to have stronger binding to TFs than those that downregulated in the MRPA (*t*-test *P* = 6.9×10^−5^). (*C*) Correlation analysis between emVar MPRA expression modulating effect (LFC cDNA:pDNA) and the predicted change in TF-binding score, noting a significant moderate correlation between (*r* = 0.3, *P* = 0.0004). In all three panels, delta (Δ) TF-binding score is calculated as the introgressed TF-binding score minus the nonintrogressed binding score.

We further found concordance between emVars and TF-binding data. We used FIMO (Grant et al. 2011) to search predicted TF-binding motifs for each introgressed and nonintrogressed sequences, filtered for TFs that are expressed in K562 cells (see Materials and Methods), and calculated a ΔTF-binding score as the predicted introgressed binding strength minus the predicted nonintrogressed binding strength. We found that emVars are more likely to lead to both large changes in predicted binding strength than either nonactive sequences (1.91, *P* = 2.1×10^−5^) or active sequences with no emVar (OR = 1.93, *P* = 2.9×10^−5^) (fig. 3*A*). Furthermore, we found that emVars that upregulated expression in the MPRA were predicted to have stronger binding to TFs than those that downregulated in the MRPA (*t*-test *P* = 6.9×10^−5^) (fig. 3*B*). Finally, we found a significant moderate correlation between emVar MPRA expression modulating effect (LFC cDNA:pDNA) and the predicted change in TF-binding score (*r* = 0.3, *P* = 0.0004) (fig. 3*C*). Overall, these results suggest that part of the mechanism by which emVars alter expression is through changes in relevant TF binding.

### emVars Influence Immune-Related TFs, Genes, and Phenotypes

We took a variety of approaches toward associating emVars and putative phenotypic effects. We examined the top ten variants with the greatest magnitude of expression modulation effect and found four predicted to alter the binding of at least one immune-related (defined as acting specifically within immune cells or in immune pathways) TF (table 1) (see Materials and Methods). Of particular note, one of these strong-acting emVars, the introgressed allele rs12534421 (C:A) is a strong up-regulator (expression modulation LFC = 3.76, *P*-adj = 0.005) and is nearest to *interferon regulatory factor 5 (IRF5)* for which it acts as an eQTL (GTEx Consortium 2015) (normalized effect size in whole blood 0.26, *P* = 2.9e-10). This gene codes for a TF active in both innate and cell-mediated responses and mutations in this gene are associated with autoimmune disorders such as inflammatory bowel disease (Dideberg et al. 2007) and rheumatoid arthritis (Rueda et al. 2006). Importantly, all but one of these extreme activating variants are at their highest frequency outside of Europe, which is the most common genetic background present in functional data sets, illustrating the value of our approach which allows us to test variants from a wide set of populations.

**Table 1.** Top 10 Most Extreme Up- and Downregulating Variants in K562 MPRA.

SNP	rs	MPRA Expression Modulation LFC	Nearest TSS	Strongest Altered TF Motifs^a^	Highest Frequency Population (Data Set)
15:85661506	rs10520585	5.11	*PDE8A*	*GATA1^1^*	32% Southwest Europe (EGDP)
5:154095231	rs17116390	4.11	*LARP1*	—	32% Northeast Siberia (EGDP)
7:128624073	rs12534421	3.76	*IRF5*	—	31% Americans (SGDP)
7:927298	rs79351189	3.31	*GET4*	*NRF1^2^*	37% Central Siberia (EGDP)
6:52710982	rs78882075	3.15	*GSTA5*	*KLF9^3^*	23% South Asia (EGDP)
1:37639441	rs74703550	−2.46	*GRIK3*	—	48% Melanesia (SGDP)
2:173533322	rs16860789	−2.87	*RAPGEF4*	—	24% East Asia (1KG)
11:460765	rs35389167	−3.05	*PTDSS2*	—	41% Americas (SGDP)
6:123112403	centered at rs13200490 with rs12524772	−3.74	*SMPDL3A*	*MEF2A^4^*	43% Americas (SGDP)
1:212255636	rs72750369	−4.09	*DTL*	—	40% Americas (SGDP)

a The references provided for each TF show provide evidence of immune function: ^1^Nei et al. (2013), ^2^Suliman et al. (2010), ^3^Yu et al. (2019), ^4^Pan et al. (2004).

**Fig. 4.**
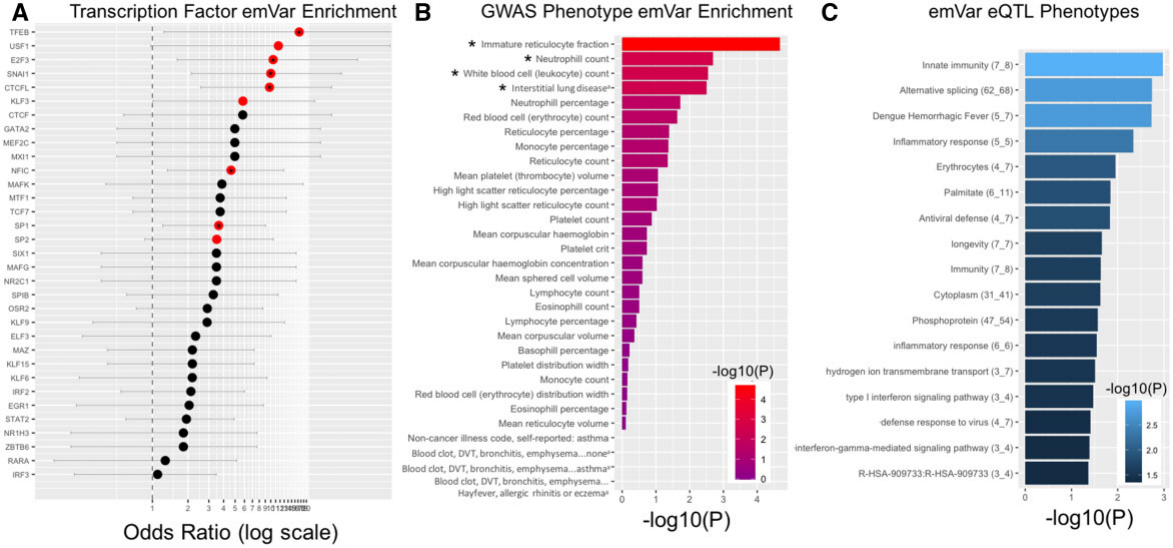
Phenotypic analysis of emVars. (*A*) Transcription factor-binding site enrichment analysis comparing the number of predicted changes to TF-binding motifs by emVars compared with non-emVars. Enrichments are reported as Fisher’s OR with lines indicating confidence intervals. Significant enrichments (*P* < 0.05) are colored in red, those surviving FDR correction (FDR<0.1) are denoted with an asterisk. (*B*) Enrichment analysis of all tested alleles and emVars with GWAS data acquired from the Neale Labe (http://www.nealelab.is/uk-biobank/, last accessed November 19, 2021) (see Materials and Methods). Asterisks indicate four phenotypes for which emVars are significantly enriched relative to tested non-emVars after FDR correction (*P* < 0.05, FDR<0.05). (*C*) Enrichment analysis using DAVID (Huang et al. 2009a, 2009b) of emVar response genes determined via eQTL analyses in immunologically relevant cell lines. The background set is the entire human genome. A number of highly relevant immunological phenotypes are observed as significantly enriched. The number of response genes and emVars is denoted next to each phenotype in the form “Phenotype Name (#Genes_#emVars).”

We next looked en masse at the predicted changes to TF binding using a hypergeometric test to examine which TFs are enriched for having binding motifs affected by emVars compared with non-emVars (fig. 4*A*). We saw overrepresentation of emVars in several TF-binding motifs including six that were significantly enriched (*P* < 0.05) and survived FDR correction (FDR<10%): TFEB (OR = 17.4), E2F3 (OR=10.5), SNAI1 (OR=10.0) CTCFL (OR = 9.76), NFIC (OR = 4.6), SP1 (OR 3.6). Several of these TFs have important immune functions. For example, TFEB, the TF for which emVars are most overrepresented, is a master regulator of lysosomal biogenesis and autophagy (Settembre et al. 2011) and has been shown to mediate cytokine and chemokine expression in immune stimulated macrophages (Visvikis et al. 2014; Pastore et al. 2016). SP1 is critical to the immune system as overexpression of this TF has been shown to activate the OAS-RNase L innate immune antiviral pathway (Dupuis-Maurin et al. 2015). E2F3 and SNAI1 have both been shown to be associated with immune infiltration in various diseases (Trikha et al. 2016; Deng et al. 2020; Fang and Ding 2020).

We also explored the distribution of emVars in GWAS phenotypes. Although we only have available GWAS associations with European populations (via the UKBiobank; Sudlow et al. 2015), it has been shown that at least a portion of intogressed GWAS associations are shared across populations (Dannemann 2021). Therefore, we examined the distribution of all tested alleles and emVars in a subset (see Materials and Methods) of UK Biobank GWAS signals (Neale Lab; http://www.nealelab.is/uk-biobank/). We found 31 phenotypes for which at least one emVar was significantly associated (*P* < 10^−7^). We conducted an enrichment analysis and found four phenotypes for which emVars are significantly enriched (*P* < 0.05, FDR < 0.05) relative to tested non-emVars: 1) immature reticulocyte fraction, 2) neutrophil count, 3) white blood cell (leukocyte) count, and 4) noncancer illness code, self-reported: interstitial lung disease (fig. 4*B*). Although immature reticulocyte fraction relates more to the effects that emVars may have on the erythrocytic properties of K562 cells, the enrichment of emVars contributing to neutrophil and white blood cell count, support a contribution of Neanderthal introgressed variants to immune phenotypes. These enrichments are driven by emVars from multiple distinct introgressed 40-kb windows with seven emVars across five windows significantly associated with neutrophil count and nine emVars across seven windows significantly associating with white blood cell count. Not all of the windows with emVars contributing to each of these phenotypes were selected in the same populations (supplementary table S7, Supplementary Material online) with, for example, in the case of the white blood cell count, one window under positive selection just in South Asia (9:112720001–112760000) with another window (10:64400001–64440000) under positive selection just in European or European-adjacent populations (Racimo et al. 2017; Jagoda et al. 2018). Although it remains to be confirmed that the association with these phenotypes holds across the non-European populations, if these associations hold, this could be an example of multiple different introgressed haplotypes contributing to the same immune phenotypes across the globe.

Finally, we examined the phenotypes associated with the response genes for the emVars that are eQTLs. Although we examined whether these eQTL emVars are enriched for any phenotypic associations compared with tested non-emVars and active sequences using a variety of gene ontology and pathway enrichment tools, we did not find any significant enrichments. Ultimately, this is to be expected because, due to the nature of positive-selection on introgressed haplotypes, each emVar is in linkage with several other non-emVar variants and the linkage ensures that they are all associated with the expression of the same genes, which is compounded by the fact that there are only 120 distinct response genes for eQTL emVars. Therefore, to ascertain the phenotypic implications of the response genes that may be affected by these eQTLs, we input the response genes into DAVID (Huang et al. 2009a, 2009b) with the entire human genome as a background set to explore the putative phenotypic consequences of the emVars, even though this does not mark an enrichment relative to non-emVars. Many of these phenotypes for which the emVar eQTLs impact constitute important immune phenotypes including innate immunity, dengue hemorrhagic fever, inflammatory response, antiviral defense, and type 1 interferon signaling pathway (fig. 4*C*). The emVars that are eQTLs for genes in these pathways constitute potential drivers for section due to their effects on these phenotypes.

### emVars as Putative Drivers of Positive Selection Signals across the Globe

In order to best hypothesize as to the putative selective and phenotypic consequences of these emVars, we examined the confluence of MPRA, eQTL, Hi-C and/or TF-binding data along with clear evidence of positive selection from population genetics studies and phenotypic associations. Below, we briefly describe three specific examples in which these data converge to suggest emVars that act as possible drivers of positive selection in immune contexts. The frequencies of all variants discussed here and in the sections below are reported in supplementary table S10, Supplementary Material online.

#### The TLR1-6-10/FAM114A1 Locus and Immune Response in Eurasian Populations (supplementary fig. 10 and supplementary table S8, Supplementary Material online)

We tested 28 linked introgressed variants that are eQTLs (supplementary fig. 10*A*, Supplementary Material online) along a haplotype that has been shown to be under positive selection across Eurasians (Sankararaman et al. 2014; Dannemann et al. 2016; Deschamps et al. 2016; Racimo et al. 2017; Jagoda et al. 2018) for *Toll-Like Receptor 1*, *6*, and *10* (*TLR1-6-10)* genes and the neighboring *FAM114A1* gene. Via the MPRA, we found support for rs73236616 as an emVar that may be driving positive selection at this locus (supplementary fig. 10*A*, Supplementary Material online). Hi-C chromatin interaction data show an interaction between the CRE containing this variant and *FAM114A1* (supplementary fig. 10*A*, Supplementary Material online), which may in some way facilitate the association between alleles at this locus and protection against various immune-related diseases including asthma and hay fever (supplementary fig. 10*C* and supplementary table S8, Supplementary Material online).

#### GMEB2 and Protection Against Gastrointestinal Diseases in Asian Populations (supplementary fig. 11 and supplementary table S9, Supplementary Material online)

We tested 16 linked eQTLs for *GMEB2* along a haplotype that has shown to have been under positive selection in Asian populations (Sankararaman et al. 2014; Racimo et al. 2017; Jagoda et al. 2018). We identified four emVars at this locus, including one, rs1304325, that also overlaps a Hi-C interaction peak for *GMEB2* (supplementary fig. 11*A*, Supplementary Material online), making it a strong candidate for driving the association between these variants and the expression of this gene (supplementary fig. 11*B*, Supplementary Material online). Given the association between this variant and protection from several autoimmune gastrointestinal diseases (supplementary fig. 11*C* and supplementary table S9, Supplementary Material online) that *GMEB2* is also associated with, this variant is a candidate driver for positive selection at this locus.

#### Severe COVID-19 Protection at the OAS1-2-3 Locus and Risk at the Chr3 CCR Cluster (supplementary figs. 12 and 13, Supplementary Material online)

Recently, two associations were observed between introgressed Neanderthal alleles and risk of severe COVID-19 response. One association is with genetic variants on chromosome 3 that exhibit significant increased risk of COVID-19 severity (Zeberg and Pääbo 2020a; COVID-19 Host Genetics Initiative 2020) and which shows evidence of positive selection in South Asian Populations (Sankararaman et al. 2014; Racimo et al. 2017; Jagoda et al. 2018). We included four variants in the COVID-19 risk locus, one of which was within an active CRE, rs71327017 (activity LFC nonintrogressed = 0.27 *P* = 0.006; activity LFC introgressed = 0.43 *P* = 5.5×10^−5^, expression modulation LFC not significant). We provide these findings for functional follow-up studies for biomedical use (supplementary fig. 12, Supplementary Material online).

The other association is with a cluster of introgressed genetic variants on chromosome 12 overlapping the *OAS1-2-3* locus that shows a protective effect against severe COVID-19 response (Zeberg and Pääbo 2020b). Although there are three missense variants at the protective locus (Zeberg and Pääbo 2020b), we explored the effects of noncoding variants as well. We tested 63 SNPs in the vicinity of *OAS1-2-3* directly falling within the severe COVID-19 protective locus in strong LD with the lead GWAS variant (*r*^2^ = 0.98; 1000 Genomes GBR) and six additional variants in moderate LD with the lead variant (*r*^2^ ≥ 0.5). Two of the strongly associated variants, both of which are eQTLs for *OAS1* and *OAS3* in multiple cell types (supplementary fig. 13*A* and *B*, Supplementary Material online), are emVars, including one, rs61478890, which falls within Hi-C interaction peaks for *OAS1* and *OAS3* (supplementary fig. 13*A*, Supplementary Material online). This variant appears to drive expression modulation via altering the TF-binding motif of SPIB (supplementary fig. 13*C*, Supplementary Material online). Because of these multiple lines of evidence for the functional effect of this variant on these *OAS* antiviral genes which may have effects on COVID-19 phenotype, this variant is a potential driver or contributor to the protective effect of this locus.

### CRISPR-Cas9 Knock Outs Validate emVars and Identify Response Genes

**Fig. 5.**
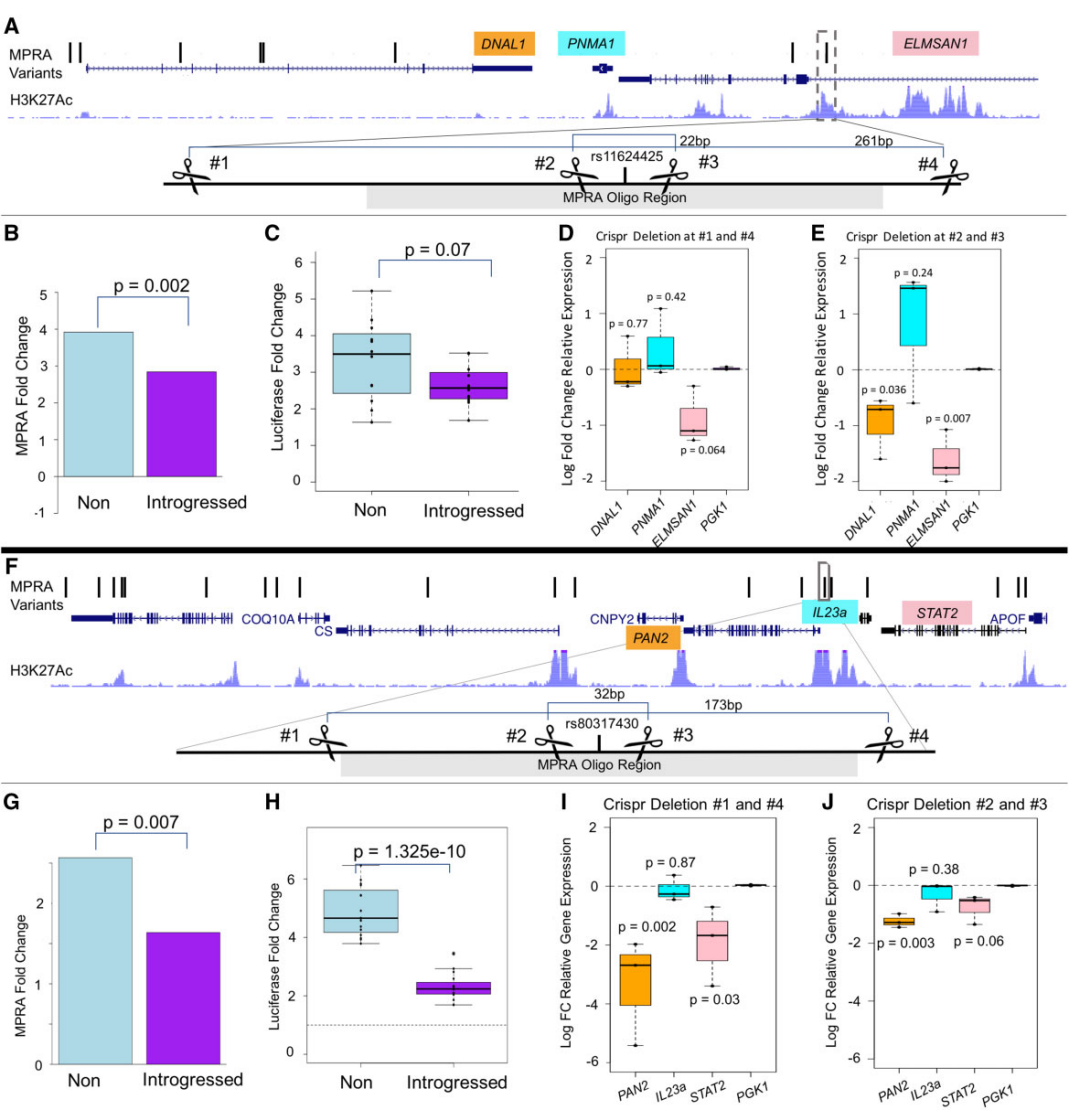
Functional experiments on rs11624425 and rs80317430 emVars. (*A*) rs11624425 locus plot and CRISPR-cas9 targeting strategy. All eight MPRA variants associated with *PNAM1* expression are plotted as ticks along a minimal schematic of the region chr14-74,096,432–74,265,785 with the three genes of interest noted. The location of the region surround rs11624425 is boxed and the below schematic indicates the relative location of the crispr guides and the sizes of the two deletions created. (*B*) rs11624425 MPRA activity results. (*C*) rs11624425 SLRA results. (*D*, *E*) qPCR results for CRISPR-deleted cells surrounding rs11624425. (*F*) rs80317430 locus plot and CRISPR-cas9 targeting strategy. The location of the region surround rs80317430 is boxed within the minial schematic of the region chr12:56,695,000–56,765,000 with the genes of interest noted. The other two *Il23a*-associated MPRA tested variants fall outside the bounds of this graph. The below schematic indicates the relative location of the CRISPR guides and the sizes of the two deletions created. (*G*) rs80317430 MPRA activity results. (*H*) rs80317430 SLRA results. (*I*, *J*) qPCR results for CRISPR-deleted cells surrounding rs80317430.

We next performed follow-up analyses for variants at loci with strong evidence of positive selection, immune relevance, and that were determined to be expression modulating both in the MPRA and SLRA experiments. In the SLRA experiments, we tested and validated as emVars variants that are eQTLs for many important immune genes including: *IL18 (rs5744258:C:G)*, a pro-inflammatory cytokine (Dinarello et al. 2013), as well as *ASB5* (rs13129964:G:A) and *FBXO7* (rs73170400:A:C), two genes in the MHC-mediated antigen processing and presentation pathway (Jassal et al. 2020) (table 2). Given their support in both assays and that they are eQTLs for genes in important immune pathways, these are good candidates for future functional testing. We also doubly validated variants at the *PNMA1/ELMSAN1* and *IL23A/STAT2/PAN2* loci, as described below (fig. 5).

#### Analyses of the PNMA1/ELMSAN1 Locus on Chromosome 14

In the MPRA, we tested eight linked variants on chromosome 14 (minimum r^2^ for any two variants is 0.85 for 1KG Europeans) (fig. 5*A*), which have been previously shown to reside in an introgressed haplotype (Quach et al. 2016). This haplotype is at its highest frequency in SGDP Americans (31%), is in high frequency in Europeans (e.g., 29% in 1KG Europe; 27% in SGDP West), but is virtually absent in East Asia (supplementary table S10, Supplementary Material online), suggesting regionally specific positive selection. Furthermore, this haplotype overlaps two 40-kb windows with strong evidence of positively selected introgression in EGDP East Northeast European and Volga-Ural populations (Jagoda et al. 2018). The MPRA found that three of these variants are emVars, one of which, rs11624425, was also validated in the SLRA assay (MPRA expression modulation LFC = −0.46 *P* = 0.002; SLRA expression modulation LFC = −0.2 *P* = 0.07, fig. 5*B* and *C*). This variant is an eQTL for two genes with known immune functions: *PNMA1*, a gene that stimulates interferon expression and physically interacts with the H1N1 virus in infected individuals (Shapira et al. 2009), and *ELMSAN1* (also known as *MIDEAS* and *C14orf43*), a TF expressed in a wide range of tissues (GTEx Consortium 2015) that has been associated with differential levels of Vaccinia virus infection (Sivan et al. 2013) and decreased IL-8 secretion (Warner et al. 2014). *ELMSAN1* is also significantly downregulated in response to influenza virus A (IAV) infection (Quach et al. 2016), and is associated with immune phenotypes including “eosinophil percentage of white cells” (*P* = 4.77×10^−9;^Astle et al. 2016) and “neutrophil percentage of granulocytes” (*P* = 1.13×10^−8^; Astle et al. 2016).

To identify the target gene(s) of emVar rs11624425, we used combinations of CRISPR-Cas9 sgRNA vectors to excise from the endogenous genome of K562 cells a 261-bp region containing the MPRA-tested 170-bp CRE and a smaller 22-bp region just surrounding rs11624425. We then used qPCR to determine the effect that these deletions had on the expression of putative response genes relative to a housekeeping gene (see Materials and Methods). Consistent with Hi-C interaction data showing an interaction between this variant and the promoter of *ELMSAN1* in immune cells (Jung et al. 2019), the most significant effect of both deletions was on *ELMSAN1* which displayed a reduction in expression that trended toward significance with the larger deletion (LFC = −0.89 and *P* = 0.064) and was significant with the smaller targeted emVar deletion (LFC = −1.61 and *P* = 0.007) (fig. 5*D* and *E*). With the smaller targeted deletion, *DNAL1* also showed a reduction in expression (LFC = −0.95, *P* = 0.036) (fig. 5*E*), though to a lesser degree. Given that the introgressed variant “G” at rs11624425 reduces the activity of the enhancer region in the MPRA and SLRA (fig. 5*B* and *C*), these results suggest that the reduction in expression of *ELMSAN1* driven by the introgressed allele may be a possible driver of positive selection at this locus. Importantly, ChIP-seq experiments (Sloan et al. 2016) demonstrate that E2F6, a transcriptional repressor (Trimarchi et al. 1998) is biochemically bound at this variant location in K562 cells with the introgressed allele predicted to increase the binding of this repressor (Ward and Kellis 2012). This suggests a mechanism of action for this reduction in enhancer activity. Overall, we hypothesize that this variant may help facilitate immune response by further decreasing *ELMSAN1* expression in accordance with the immune response. We note that more work is needed to understand the immune significance of *ELMSAN1* and the phenotypic effect of rs1164425.

#### Analyses of the IL23A/STAT2/PAN2 Locus on Chromosome 12

We next explored a region on chromosome 12 from which we tested 20 linked alleles in the MPRA (fig. 5*F*). This haplotype was previously shown to be under positive selection in Melanesian populations (Mendez et al. 2012). These alleles are all at extremely high frequency (55%) in SGDP Melanesians and EGDP Oceanic individuals (50%), while present but at much lower levels in other populations (e.g., 7% in 1KG Europeans) (supplementary table S10, Supplementary Material online). Three tested variants were shown to be emVars, one of which was further validated in the SLRA, rs80317430 (MPRA expression modulation LFC = −0.65 *P* = 0.007; SLRA LFC = −0.76 *P* = 1.3×10^−10^, fig. 5*G* and *H*; table 2). The variant rs80317430 is an eQTL for many genes (GTEx Consortium 2015), including *IL23A*, a pro-inflammatory cytokine, *STAT2*, a critical part of the interferon immune signaling response for which our emVar set is enriched (see above), and *PAN2*, a part of the poly(A)-nuclease deadenylation complex which deadenlyates mRNA leading to its degradation (Wolf and Passmore 2014). Although this allele falls within the 5′-UTR of *PAN2*, ChIP-seq studies indicate the region may also play a direct role in transcriptional regulation (Sloan et al. 2016). For example, many TFs are bound to the region in K562 cells (Ward and Kellis 2012) (see Materials and Methods). We used CRISPR-Cas9 editing in K562 cells to excise a 173-bp region containing the entire active CRE sequence and a smaller targeted 23-bp deletion immediately surrounding emVar rs80317430 (fig. 5*A*). Both deletions significantly reduced *PAN2* expression (173 bp deletion: LFC = −3.36, *P* = 0.002; 22 bp deletion: LFC = −1.24, *P* = 0.003) and, to a lesser degree, *STAT2* expression (173 bp deletion: LFC = −1.93, *P* = 0.03; 22 bp deletion: LFC = −0.76, *P* = 0.03) (fig. 5*I* and *J*) indicating that these two genes are regulated by the *cis*-regulatory region containing rs80317430.

Both *STAT2 and PAN2* are upregulated in response to IAV infection (Quach et al. 2016). However, hyperactive *STAT2* expression can lead to lung damage during infection, including most recently from *SARS-CoV-2* infection (Boudewijns et al. 2020). Thus, if the introgressed allele at rs80317430 mediates a decrease in *STAT2* expression as predicted by both MPRA and SLRA, it could be beneficial in protecting against this damaging immune response. *PAN2* also regulates *HIF-1a* mRNA stability (Bett et al. 2013), which is critical to hypoxic response and in immune pathways (Palazon et al. 2014), but can also be protumerogenic (Bett et al. 2013), suggesting another benefit to its decreased expression. Finally, *PAN2, STAT2*, and rs80317430 are all significantly associated with mean platelet volume (*PAN2 P* = 2.67×10^−10^, *STAT2 P* = 3.33×10^−10^; Astle et al. 2016) with the introgressed “A” being associated with a decrease in mean platelet volume (*P* = 2.98 10^−8^; Astle et al. 2016). As mean platelet volume is positively associated with stroke risk (*P* = 0.01) (Bath et al. 2004), an allele that decreases MPV could also be indicative of protection from stroke risk.

## Discussion

DNA sequence analysis has shown that modern and archaic humans interbred. Since then, one of the big remaining mysteries is characterizing the function and impact of archaic alleles in modern humans. Here, we identified 292 Neanderthal introgressed alleles that have reached appreciable frequencies in modern human populations, and which show direct evidence of having a regulatory effect in K562 cells. We also identified 2,548 such variants that fall within active CREs in this cell line. By specifically investigating the effects of each Neanderthal allele independently, we are able to identify putative driver variants of positive selected introgressed haplotypes to connect putative drivers to changes in gene expression, TF-binding and phenotypic associations. This work adds a useful data set to a growing effort to tease out the effects of introgressed variation on gene expression and phenotypic variation and to disentangle the phenotypic contribution of introgressed variation from linked human alleles (Skov et al. 2020).

MPRA experiments are becoming a new tool in the effort to use genetics to understand evolutionary questions. For example, MPRAs have been conducted looking at the effects of fixed differences between humans and Neanderthals/Denisovans (Weiss et al. 2021) as well as regions of the human genome that have been largely diverged since the split from chimpanzees (Uebbing et al. 2021). These studies have produced rich data sets for additional research and have produced interesting findings, such as the finding that variants that are fixed between humans and Neanderthals/Denisovans are enriched for functionality in the vocal tract (Weiss et al. 2021). Our work here is the first MPRA study to specifically investigate archaically introgressed alleles and we hope that the contribution of this data set to this growing list of emVars in human populations aids in future evolutionary studies. Notably, we find that a smaller percentage of the variants within active elements are emVars in our introgressed set (11.5%) compared with 30% of variants in active elements from human-accelerated regions (HARs) and human gain enhancers (HGEs) (Uebbing et al. 2021), and 30% and 25% of human fixed differences tested in osteoblasts and neural progenitor cells, respectively (Weiss et al. 2021). However, in this latter study the authors did identify only 9% emVars in embryonic stem cells. Though a more thorough comparison of the results across these studies should be undertaken, this result could be consistent with the majority of retained Neanderthal introgressed alleles being tolerated because they are less likely to have a functionally different effect than alleles that remained fixed between humans and Neanderthals despite introgression. Another consideration of the relationship between this MPRA and previous efforts is the relatively high-expression modulation values we detected of the positively introgressed alleles tested here. We detected 8.2% of emVars had an absolute expression modulation LFC greater than 2. In comparison, Weiss et al. (2021) detected 0–1.8% of fixed difference alleles had this strong of effect, whereas Tewhey et al. (2016) found 5.5% of tested eQTLs and Uebbing et al. found 16.8% of alleles in HARs and HGEs had this at least this strong of an effect. Whether this is due to the nature of positively selected introgressed alleles perhaps being selected for relatively strong effects relative to other types of variants, should be explored.

There are some notable limitations with MPRA in general as well as with our analyses specifically. For example, MPRA is prone to many false negatives for a variety of reasons including that variants may have expression modulating potential in a cell type not tested, and/or that such variants may reside in a CRE larger than the tested element size. Furthermore, because we used an episomal reporter vector, which has been shown to be somewhat less accurate than integrated reporter assays (Inoue et al. 2017), this may have impacted some of our results. The episomal nature of this experiment may also contribute to some issues with direction of effect for emVars as previously reported (Ip et al. 1991; Dubnicoff et al. 1997; Tewhey et al. 2016). Furthermore, direction of effect can be influenced by cell type and may be opposite even in closely related cell types, though is thought to be relatively uncommon (∼7.4% of eQTLs) (Mizuno and Okada 2019). Finally, although here we tested variants from across 20 populations in the MPRA, the interpretation of the adaptive nature of emVars from non-European populations is limited by the lack of eQTL, GWAS, and other data from non-European populations. Therefore, we stress taking a conservative approach in interpreting MPRA variant impacts and encourage the integration of MPRA data with other relevant data sets including chromatin mark, Hi-C, and TF-binding analyses in addition to follow-up experiments as we have done here.

Despite these limitations, we found that these introgressed emVars are enriched for signals of endogenous function, including overlaps with chromatin mark data and enrichment in Hi-C interactions. We further found that emVars are enriched for altering important immune TF-binding sites, are enriched for significantly associating with neutrophil and white blood cell counts, and are associated with the expression of genes that act within important immune pathways including innate immune response and antiviral response.

Although previous studies have identified introgressed haplotypes that are associated with immune-specific gene expression and pathways (Dannemann et al. 2016; Deschamps et al. 2016; Sams et al. 2016), we are here able to propose specific mechanistic explanations for some potential selective sweeps in human populations. In the case of OAS pathways, such mechanistic understanding is crucial, as introgressed variants linked to the expression of *OAS1*-*2*-*3*, including one emVar identified in the MPRA, have been found to be somewhat protective against severe COVID-19 response (Zeberg and Pääbo 2020b). Because we included variants in this assay that derived from 20 different populations, we were able to identify driver variants of signals of positive selection across the globe including for protection against gastrointestinal diseases in South Asians.

We were also able to directly link a region surrounding an introgressed allele at rs11624425 at high frequency in Europeans and people indigenous to the Americas to *ELMSAN1* expression and show that the introgressed allele reduces the activity of an enhancer for this gene. Previous work has shown that *ELMSAN1* is significantly downregulated in response to flu infection in vitro (Quach et al. 2016), suggesting that the reduced activity resulting from the introgressed allele at rs11624425 could help facilitate human flu response. This locus therefore may be an example of a previous finding that viruses, and especially RNA viruses such as flu, have been strong drivers of selection in human evolution, particularly in Europeans (Enard and Petrov 2018). Similarly, we are also able to connect a downregulating introgressed allele at rs80317430 to expression of two genes, *PAN2* and *STAT2*, both of which are upregulated in response to flu infection (Quach et al. 2016), but also can contribute to potentially damaging responses including inflammation and hypoxia response. The downregulating introgressed allele may act to mediate these effects. The allele may also contribute to decreased stroke risk via a reduction in mean platelet volume. Our targeted approach of using CRISPR-Cas9 deletion assays combined with integration of other types of data allowed us to link the tested introgressed variant(s) to these primary response genes and to generate adaptive hypotheses.

Overall, our work illustrates the ability to identify functionally relevant potential driver alleles of introgressed selective sweeps and provides 292 lab-tested candidate variants that may drive differential expression of immune genes across human populations. This study could serve as a model for identifying such drivers in the future and could be expanded to include introgressed variants relevant to other cell types and physiological functions.

## Materials and Methods

### Generation of Putatively Adaptively Introgressed Variant Set

Although Neanderthal introgression spans 1.5–2% of the genomes of all non-African living humans (Prüfer et al. 2017), it is likely that only a subset of these base pairs is actually functionally important. Therefore, we designed a variant selection scheme to identify SNPs that are most likely to have phenotypic effects. Accordingly, we first filtered for variants that show evidence of having been under positive selection in humans. Given that each individual human population is expected to have only a few true cases of positive selection from introgression (Gittelman et al. 2016), we maximized the possibility of detecting potentially adaptively introgressed regulatory variation by selecting candidate variants from a geographically diverse set of populations. To generate the initial variant list, we relied on adaptive introgressed variation maps from the 1000 Genomes (1KG), the Simons Diversity Project (SGDP), and the Estonian Genome Diversity Project (EGDP). Because we began this study prior to the publication of the Vindija Neanderthal genome (Prüfer et al. 2017)^,^ the introgression maps from which we extracted variants use the Altai Neanderthal as a reference sequence (Prüfer et al. 2014). Although the Vindija genome is closer to the introgressing Neanderthal population and therefore allows for a more complete identification of introgressed variation, introgression identified using the Altai genome still represents introgression, though an incomplete set. We chose to test variants from across all studies that met the following frequency requirements: 1) homozygous in the high-coverage Altai Neanderthal sequence, 2) at least 20% frequency in the population in which it was detected, and 3) less than 1% frequency across African individuals in the 1000 Genomes Phase 3 Panel (African Americans excluded). These filters mirror those used in the “U” statistic (Sankararaman et al. 2016) and are designed to ensure that every variant has a high probability of being both introgressed and under positive selection. This approach generated a conservative list of 62,820 variants. We next filtered this variant list to remove those whose immediate flanking region (85 bp on either side, to correspond to the MPRA oligo sequences) overlaps a genomic repetitive element as described (Benson 1999). Among other issues, repetitive elements cause difficulty in the amplification steps of the MPRA and may generate enhancer RNAs that interfere with reporter output. Ultimately, this filtering process generated 59,955 adaptive introgressed variant loci. It should be noted that this scheme is designed only to target variants likely to have been under positive selection, not any other form of selection such as stabilizing or purifying selection.

### MPRA Oligo Pool Design

As a validation of the utility of MPRA to identify functional adaptively introgressed SNPs, we chose to utilize a representative variant subset consisting of ∼10% of the total of putatively adaptively introgressed variant set. In designing the subset, we put an emphasis on assessing whether variants that had been previously annotated as eQTLs via computational approaches show increased likelihood to be functionally validated as affecting gene expression in this experimental system. Such functional validation experiments are rarely performed due to the enormous cost and labor required to validate eQTL variants using traditional methods, such as SLRA or luciferase reporter vector analysis, which require individual testing of each variant. Therefore, we included the 1,156 variants in the putatively adaptively introgressed variant set that were linked to one of the top 100 eQTLs (loci that affect gene expression) in any tissue (Dannemann et al. 2017). Additionally, another 4,299 variants that were not previously annotated as eQTLs were selected from the 59,955 variant pool using a random number generator. Variants in this set were chosen irrespective of linkage to one another and in total across all experiment sets we tested variants across 2,846 40-kb windows of the genome. These two pools of experimental variants in addition to the nonintrogressed orthologous sequences accounted for a total of 10,910 sequences, making up 90.95% of the total MPRA vector pool. Additionally, we included two sets of human variants matching the frequency of the introgressed alleles in 1000 Genomes Europeans. One of these negative sets consists of 552 variants which are both frequency matched and are located between 200 and 1,000 bp of an experimental SNP. The other set only has the frequency requirement and consists of 233 variants. We generated lists of frequency matched SNPs for each allele using the SNPsnap tool (Pers et al. 2015). To get the desired proportions, we randomly sampled the experimental variant pool to get 552 and 233 variants and then input these variants into the SNPsnap tool with the appropriate parameters (max frequency difference ±5% for both sets and distance threshold for the location matched set.). For each input SNP, we retrieved 5,000 matched SNPs of which we randomly selected one to include in the analysis. We further included a positive control set consisting of 301 oligo sequences that have regulatory activity via published MPRA experiments on the similar cell lines, K562 cells (Ulirsch et al. 2016) and LCL cells (Tewhey et al. 2016). These positive control sequences were originally either 145 or 150 bp, respectively, so they were extended to 170 bp to match the other oligos in this pool by adding flanking sequence to either side, keeping the original oligo at the center of the sequence. In total, the oligo pool consisted of 11,996 sequences The proportions of each of the control sets are in accordance with Tewhey et al. (2016).

### Generation of MPRA Vectors

Following Tewhey et al. (2016), for each SNP in the MPRA adaptive introgressed variant set, two experimental reporter vectors were created, one with the adaptively introgressed variant and one with the alternative nonintrogressed variant. For each of these variants, 200 bp single-strand oligo sequences were synthesized by Twist-Bioscience. These oligo sequences contain the introgressed or nonintrogressed variant along with 85 bp of flanking sequence on either side for a total of 170 bp unique sequence per oligo. To ensure that variants are tested in the relevant functional context, we include in the oligo SNPs that fall within the flanking region that are in strong LD with the introgressed variant (*r*^2^ ≥ 0.9). This sequence is then considered as the “LD background” for the variant. There were 450 such cases in the data set accounting for 8.4% of oligo sequences. Similarly, in the nonintrogressed vector, if a nonreference allele in the flanking region is linked (*r*^2^ ≥ 0.9) in Africans to the nonintrogressed allele, the linked allele is used in the oligo. Additionally, on both sides of the oligo, 15 bp of adaptor sequence was included to facilitate cloning into the MPRA vector. All cloning steps to create the MPRA library followed Tewhey et al. (2016). Barcode-tag sequencing was performed using the Illumina NovaSeq sequencer at the Broad Institute.

### MPRA Transfections and RNA Collection

For K562 cells, for each of four replicates, 60 million cells per replicate were transfected with 60ug the MPRA library using electroporation with the Neon transfection system following the manufacturers protocol. About 24 h after transfection, cells were collected and snap frozen in liquid N_2_. Total RNA was extracted and GFP mRNA was isolated and prepared for sequencing following Tewhey et al. (2016). Sequencing was performed via 1×50 bp chemistry on an Illumina HiSeq at the Harvard Bauer Core.

### MPRA Data Analysis

All MPRA data analysis followed Tewhey et al. (2016) including methods for establishing barcode and oligo sequences links from the plasmid (DNA) sequencing data and extracting barcode counts from the RNA sequencing data.

#### Barcode–Oligo Reconstruction

The 150-bp paired end reads from the sequencing of the mpraΔorf library were merged using Flash v.1.2.11 (Magoč and Salzberg 2011). Merged amplicon sequences were then filtered for quality control such that sequences were kept if: 1) there was a perfect match of 10 bp on the left or right side of the barcode, 2) the 10 bp on both sides of the barcode matched with levenshtein distance of 3 or less, and 3) the 2 bp on either side of the barcode matched perfectly. Sequences that passed through these filters were aligned back to the expected sequence pool using Bowtie2 v. 2.3.4.1 with the –very-sensitive flag. Alignments that had less than 95% perfect matching with the expected sequence and any alignment which had a mismatch at the variant position were removed. Barcodes that matched to more than one expected sequence are unusable and therefore were also removed.

We identified an average of 13,513 unique barcodes per oligo in the plasmid sequencing and all oligos were represented in the barcode-tag sequencing. This high number of barcodes is beneficial because it represents more unique molecules throughout the entire MPRA process, including reduced PCR bias in the presequencing amplification steps. Furthermore, because, as described below, our workflow involves summing the counts across all barcodes for a single oligo sequence, a large number of different barcodes leads to reduced impact of a biologically impactful barcode affecting the transcript count.

#### Tag Sequencing

Again following Tewhey et al. (2016), the 1×50 bp tag sequencing reads were filtered such that reads were only kept if they had a maximum levenshtein distance of 4 with the constant sequence within the 3′-UTR of the GFP as well as a perfect match with the 2 bp adjacent to the barcode. If the sequence passed through these filters, the barcodes were then matched back to the oligos based on the information from the mpraΔorf library sequencing described above. The number of barcodes per oligo retained in the cDNA and pDNA is smaller than in the overall pool because of the extremely large overall diversity of the original plasmid pool. The average number of barcodes per oligo in each cDNA replicate is as follows—111.6, 111.96, 70.76, 111.49 and for each pDNA replicate 244.5, 242.9, 251.9, 224. The counts for each barcode were summed for each oligo. This summation of the counts per barcode reduces the noise that could be derived from any individual barcode having a functional effect.

#### Determination of Active Putative CREs and emVars

Following Tewhey et al. (2016), the summed oligo counts from the tag sequencing for all four cDNA samples and all four plasmid samples were passed in Deseq2 and sequencing depth was normalized using the median-of-rations method (Love et al. 2014). We then used Deseq2 to model the normalized read counts for each oligo as a negative binomial (NB) distribution. Deseq2 then estimates the variance for each NB by pooling all oligo counts across all the samples and modeling the relationship between oligo counts and the observed dispersion across all the data. It then estimates the dispersion for each individual oligo by taking this observed relationship across all the data as a prior to performing a maximum posteriori estimate of the dispersion for each oligo. Therefore, the bias for the dispersion estimate for each oligo is greatly reduced because it relies on pooled information from all other oligos. The correlation between normalized counts across the four replicates is extremely high (Pearson’s *r* > 0.99, *P* < 2.2e-16 across all pairwise replicates; supplementary fig. 3, Supplementary Material online), reflecting the consistency in the data that comes from summing across many unique barcodes. We then used Deseq2 to estimate whether an oligo sequence had an effect on transcription by calculating the LFC between the oligo count in the cDNA replicates compared with its count in the plasmid pool. We tested whether this LFC constituted a significant difference of expression (whether the LFC [cDNA:pDNA] significantly differs from 0) using Wald’s test and required a stringent Bonferroni corrected *P* value of less than 0.01 for a significant result. If an oligo sequence had a significant LFC with either allele, the sequence is considered “active.” As further assurance as to the precision of this measurement, we examined the ratio of cDNA counts to pDNA counts across all the replicates and again found extremely high magnitude and significant correlations (minimum Pearson’s *r* between replicates = 0.96, *P* < 2.2e-16 across all comparisons, supplementary fig. 4, Supplementary Material online).

Finally, to determine which variants are expression modulating (emVars), for oligos which were determined to be active, we used Deseq2 to calculate the fold change between the two versions of the oligo sequences with Wald’s test to calculate the *P* values. *P* values were then corrected using the Benjamini–Hochberg test to correct for multiple hypothesis testing. Significance was defined stringently as a multiple hypothesis corrected *P* value of <0.01.

For quality assurance, we examined the coefficient of variation (CV) for the normalized transcript count for every oligo and how CV is related to number of barcodes as well as how it affects the likelihood of being determined by Deseq2 as active and expression modulating. As shown in supplementary fig. 6*A*, Supplementary Material online, there is a highly significant, but moderate in magnitude, negative correlation between average unique barcode count across replicates and CV of normalized transcript count (Pearson’s *r* = −0.21, *P* < 2.2e-16), with the majority of oligos having a relative low CV (65% CV <0.3; 83.5% CV<0.5%), overall mean = 0.38, and median = 0.21. Importantly, as described above, Deseq2 appropriately accounts for dispersion, as oligos with higher CV require greater magnitude LFC cDNA:pDNA to achieve the *P* value for significance (supplementary fig. 6*B*, Supplementary Material online). This is additionally reflected in significantly lower average CV for emVars (0.15) and active non-emVars (0.18) than nonsignificantly active sequences (0.53) (*t*-test emVars vs. nonactive and active non-emVars vs. nonactive *P* value <2.2e-16; *t*-test emVars vs. active non-emVars *P* value = 5.1e-8) (supplementary fig. 6*C*, Supplementary Material online). This reflects that Deseq2 accounts for variation between replicates and shows that the significant oligos show high consistency across replicates.

### TF-Binding Analysis

For the TF-binding analysis, we downloaded the entire set of the high-quality “core collection” human transcription-binding motifs from HOCOMOCOv11 (Kulakovskiy et al. 2017). We trimmed all tested sequences to 40 bp centered around the central variant and used FIMO (Grant et al. 2011) to map the motifs to the sequences. We then removed from the analysis any TF not minimally expressed in K562 cells (FPKM <1 from K562 polyA mRNA RNA-seq GEO Accession: GSE90236; Encode Project Consortium 2012). We then computed the predicted differential binding score for each oligo pair by identifying all motifs for which either the introgressed or nonintrogressed sequence had a FIMO *P* value *P* < 10^−4^; if the other sequence had a least a *P* value *P* < 10^−3^, we computed the differential predicted binding score by subtracting from the score of the introgressed allele the score for the nonintrogressed allele (introgressed score–nonintrogressed score). If the other allele did not have a score *P* < 10^−3^, we conservatively did not include this motif in the analysis because we cannot be confident as to the binding difference. For each pair set, if multiple significant differences existed, we chose the motif with the highest absolute predicted binding difference. For the enrichment plot in figure 4*A*, we compared the number of non-emVars and emVars that altered the binding of each TF using Fisher’s exact test. *P* values were corrected for multiple hypothesis testing using the Benjamin–Hochberg method.

### GWAS Enrichments

UKBiobank GWAS data were downloaded from the round 2 Neale Lab results (http://www.nealelab.is/uk-biobank/). To reduce the number of tests, we downloaded a subset of relevant phenotypes (shown in supplementary table S11, Supplementary Material online), including all disease phenotypes, all blood-related phenotypes, and all infection-related phenotypes. We then calculated the number of variants of all classes that were significantly associated with each phenotype (*P* < 10^−7^) and compared the number of emVars with non-emVars using Fisher’s exact test. *P* values were corrected for multiple hypothesis testing using the Benjamin–Hochberg method. Overlaps with positive selection windows were performed using *Table S1* from Jagoda et al. (2018), which also includes data from Racimo et al., (2017), Sankararaman et al., (2014), and Sankararaman et al., (2016). For individual variant analyses, we sometimes supplemented this data with the full set of GWAS signals detected for a variant, which we extracted from the GWAS Atlas (https://atlas.ctglab.nl/PheWAS, last accessed November 19, 2021). These instances are noted in the text.

### Phenotype Analysis

The reported phenotype analysis shown in figure 4*C* was conducted as follows. emVars were first matched to response genes using GTEx eQTL data for whole blood, spleen, and EBV-transformed lymphocytes. Next, all emVar response genes were input in DAVID (Huang et al. 2009a, 2009b) with the human genome as the background. We downloaded the enrichment results for the following types of analysis: “UP_KEYWORDS,” “GOTERM_BP_DIRECT,” “GAD_DISEASE,” “REACTOME_PATHWAY,” “KEGG_PATHWAY.” We then removed any enrichments that were by less than three response genes.

### Promoter Interaction with emVars in Immune Tissue Hi-C Data

To identify promoters physically interacting with emVars or the CREs containing emVars, we intersecting the 170-bp CREs containing emVars with significant promotor–DNA interactions (*P* < 0.01) from Hi-C experiments on lymphoblasts, spleen, and thymus (Jung et al. 2019). We then took this list of genes whose promoters interact with emVar CREs and input them into the Reactome pathway overrepresentation analysis tool described above. Significance in this tool is defined as adjusted *P* value less than 0.05. As an additional control, we calculated a second FDR by taking all promoter interactions from the same Hi-C tissues sets and made 100 randomly generated subsets of the same size as the emVar interaction set (*n* = 221) using a random number generator and then input these 100 sets into Reactome. We then removed any enrichments that were significantly enriched in five or more random subsets for an FDR <5%.

### Single-Locus Reporter Assay

Reporter vectors were synthesized by Genewiz consisting of the 170-bp MPRA oligo cloned into the Pgl4.23 Luciferase Vector. Vectors were electroporated into DH10B *E. coli* and ultimately grown in 150 ml LB for 9–12 h to be isolated via Zymo Maxiprep kit. For each replicate for each vector, four wells of a 24-well plate were seeded with 1×10^5^ K562 cells and transfected with 500 ng of the experimental luciferase vector and 30 ng of the Pgl.4.74 renilla luciferase vector using lipofectamine 3000. For each experiment, four wells were also transfected with an empty Pgl4.23 control. About 24 h later, the cells were collected and frozen. Cells were lysed and luciferase fluorescence was activated using the Promega Dual Glo System and luciferase values were measured on a Biotek Synergy Neo2. Firefly luciferase values for each vector were normalized to the renilla firefly expression and averaged across the four wells. Results for each vector were reported as the average fold change of the normalized luciferase results of the vector relative to the average normalized luciferase value of the empty vector. Three biological replicates were performed for each vector.

### CRISPR-Cas9 Experiments

Guides were designed using the Broad Institute GPP sgRNA Designer. Forward and reverse guides were ligated and cloned into the Px458 CRISPR vector following (Ran et al. 2013). pSpCas9(BB)-2A-GFP (PX458) was a gift from Feng Zhang (Addgene plasmid No. 48138; http://n2t.net/addgene:48138, last accessed November 19, 2021; RRID: Addgene_48138). Once cloned, vectors were transfected into NEB5 alpha *E coli.* and grown to transfection concentration in 150 ml LB for 9 h. Vectors were isolated using Zymo Maxi Prep kits. For each replicate, 20ug of each vector in the desired guide combination were transfected into 10 million K562 cells using the Lonza Nucleofection system. In addition, for each replicate, 10 million cells were also transfected with 40 μg of the empty Px458 vector as a control. About 48 h later, experimental and control cells were collected and FACs sorted for GFP. About 1×10^5^ of the sorted cells for each sample were used to collected gDNA using the Omega E.Z.N.A Tissue DNA kit. Deletions were confirmed via gel or amplicon sequencing depending on the size of the deletion. Total RNA was collected from the remaining cells using the Qiagen RNAeasy mini kit. cDNA was synthesized using the Superscript III kit and qPCR was performed in triplicate. Expression was normalized to the *PGK1* housekeeping gene using the ΔΔCt method. qPCR values are reporter as the fold change of the normalized expression of the gene in the deleted cells relative to the normalized expression of the empty CRISPR vector transfected cells.

## Supplementary Material

Supplementary data are available at *Molecular Biology and Evolution* online.

## Supplementary Material

msab304_Supplementary_Data
