# Supplementary material for: Detection of Neanderthal Adaptively Introgressed Genetic Variants That Modulate Reporter Gene Expression in Human Immune Cells
Source: Mol Biol Evol. 2021 Oct 18;39(1):msab304. doi: 10.1093/molbev/msab304 (PMC8760939; doi:10.1093/molbev/msab304)
Supplement: msab304_Supplementary_Data [file msab304_supplementary_data.zip › Supplementary Information MBE Revision.pdf]

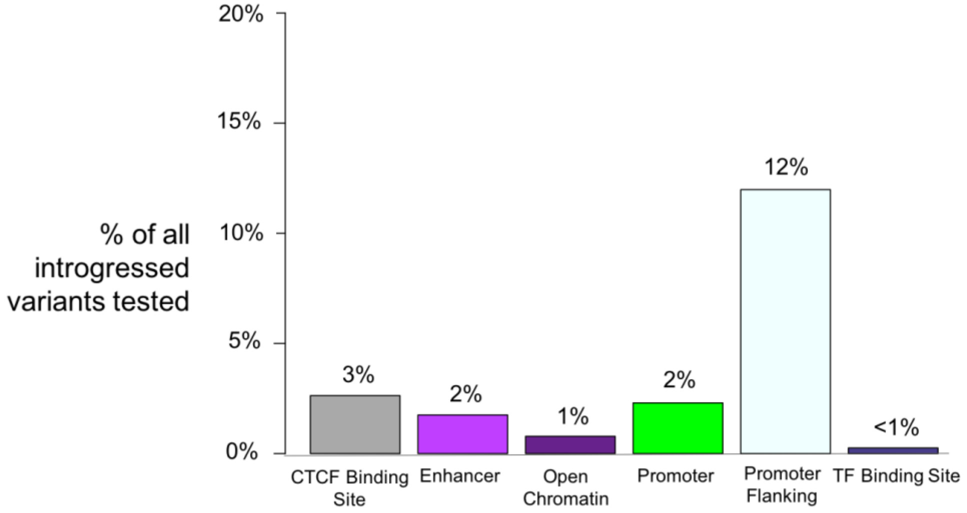

Supplementary Figure 1. Distribution of MPRA variants across Ensembl K562 features. Feature names are as defined by Ensembl (Hunt et al. 2018). Notably, “transcription factor binding sites” are defined as “sites which bind transcription factors, for which no other role can be determined as yet”.

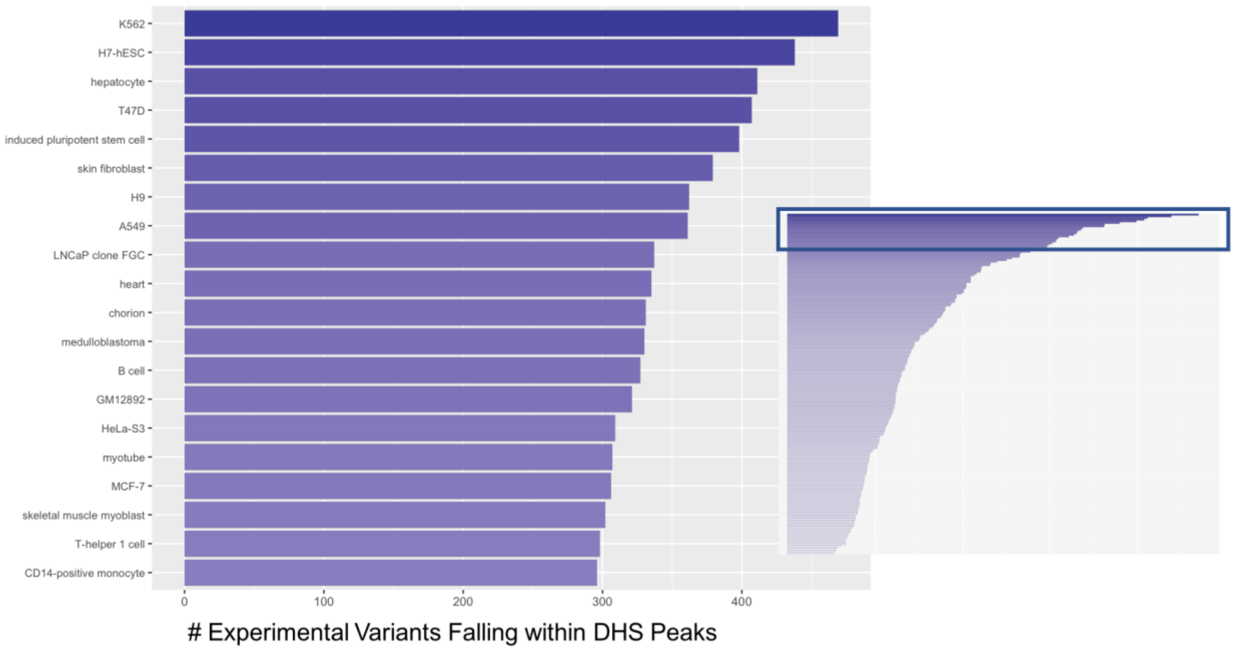

Supplementary Figure 2. Number of tested variants within ENCODE DHS peaks across cell lines/tissues. To determine which cell line was most appropriate for the MPRA we intersected our experiment set of 5,353 variants with all DHS peak files from ENCODE (Encode Project Consortium 2012) representing 738 experiments conducted across 208 unique cell lines/tissues. We then collapsed all intersections for a given cell lines/tissues and determined the total number of variants falling within a DHS peak in that cell lines/tissues in any experiment. The full distribution of these intersection counts is shown in the right insert with the top 20 cell lines/tissues displayed to the left. Each row represents a cell line or tissue. Most variants (469) fell within DHS peaks in K562 cells, so this line was chosen for the MPRA.

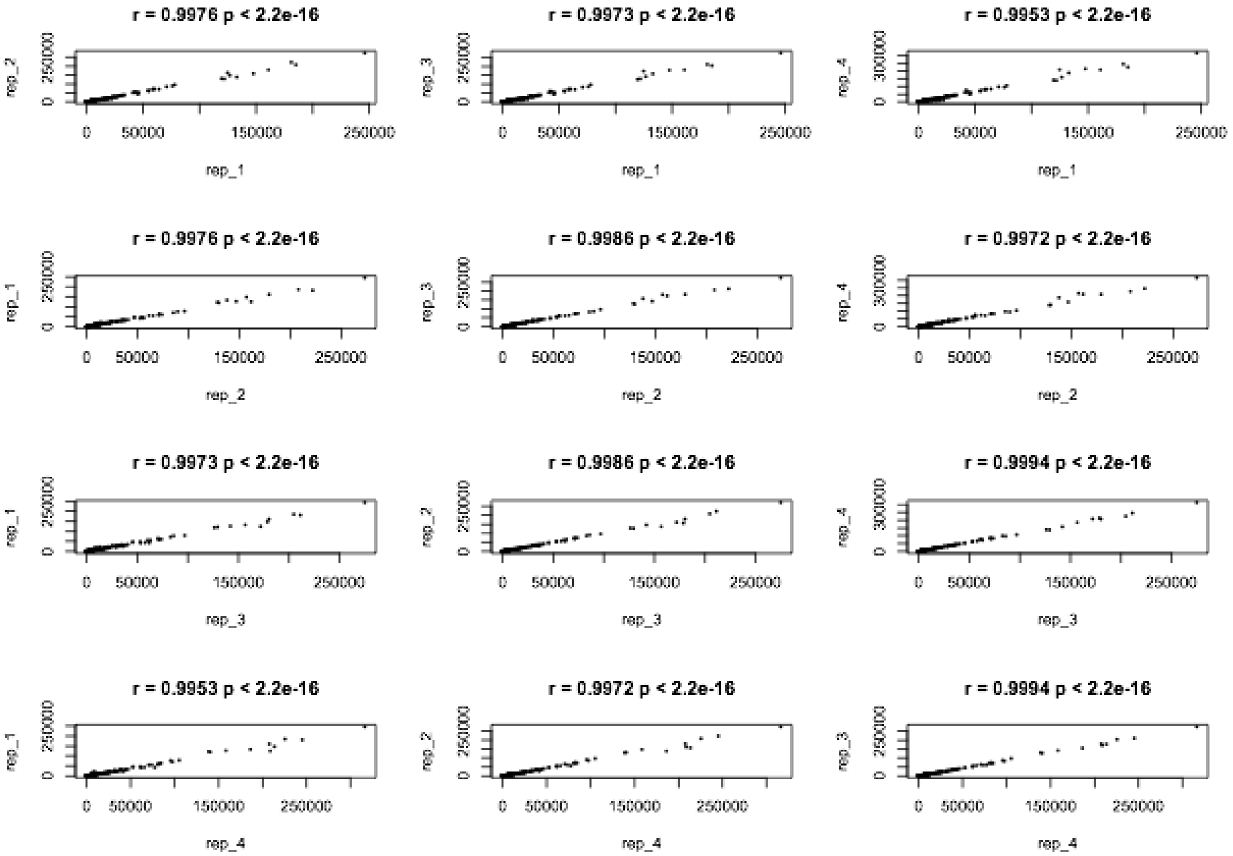

Supplementary Figure 3. MPRA normalized transcript count across experiments. Each graph compares the normalized RNA transcript counts driven by each tested element between 2 of the 4 experiments (denoted K_1, K_2, K_3, K_4). Pearson's R and associated p--value are reported above each graph.

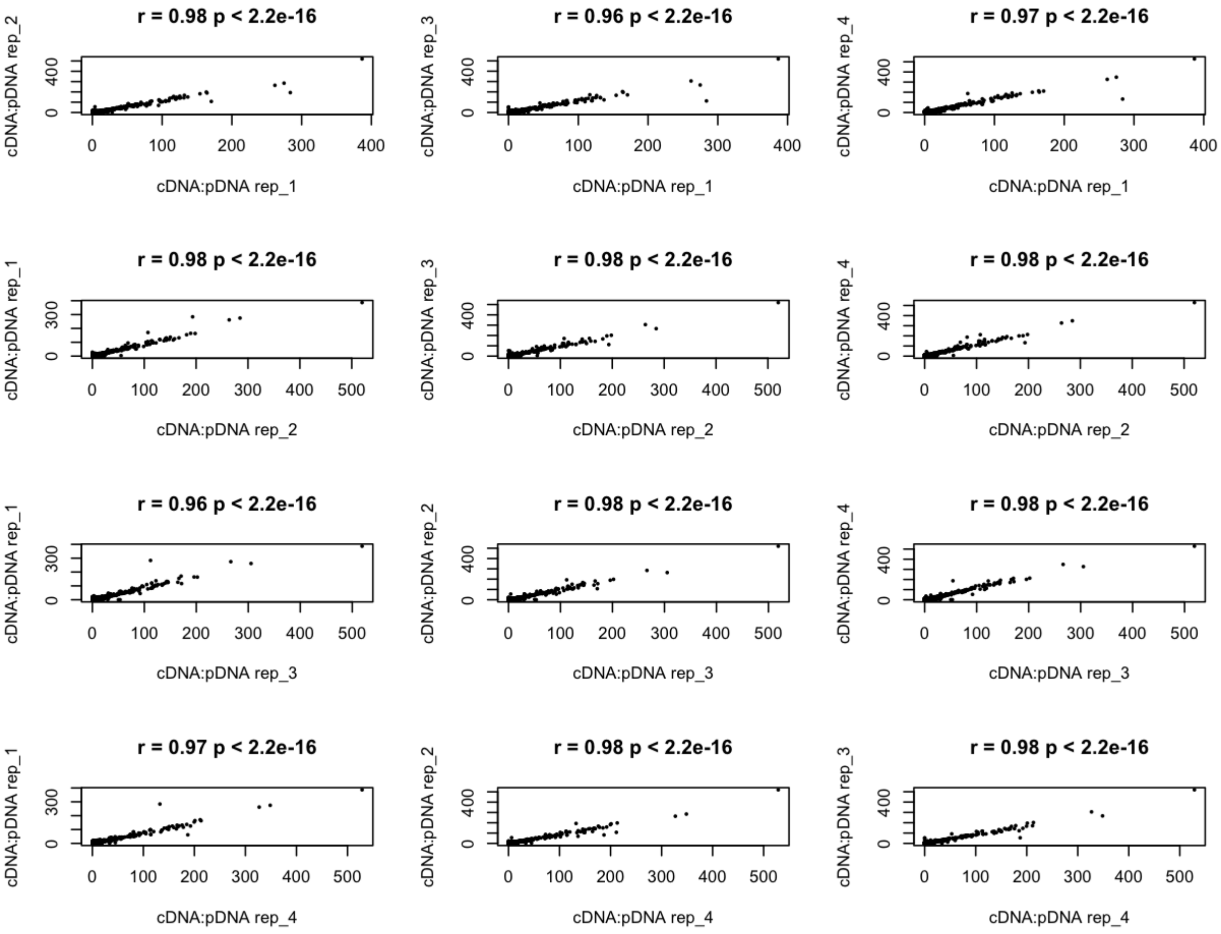

Supplementary Figure 4. The ratios of cDNA:pDNA per tested element across replicates. Each graph compares the normalized ratio of cDNA counts to pDNA counts from each tested element between 2 of the 4 experiments (denoted K_1, K_2, K_3, K_4). Pearson's R and associated p--value are reported above each graph. The ratios of cDNA:pDNA per tested element were highly reproducible across replicates (minimum r = 0.96, $p < 2.2 * 10^{-16}$).

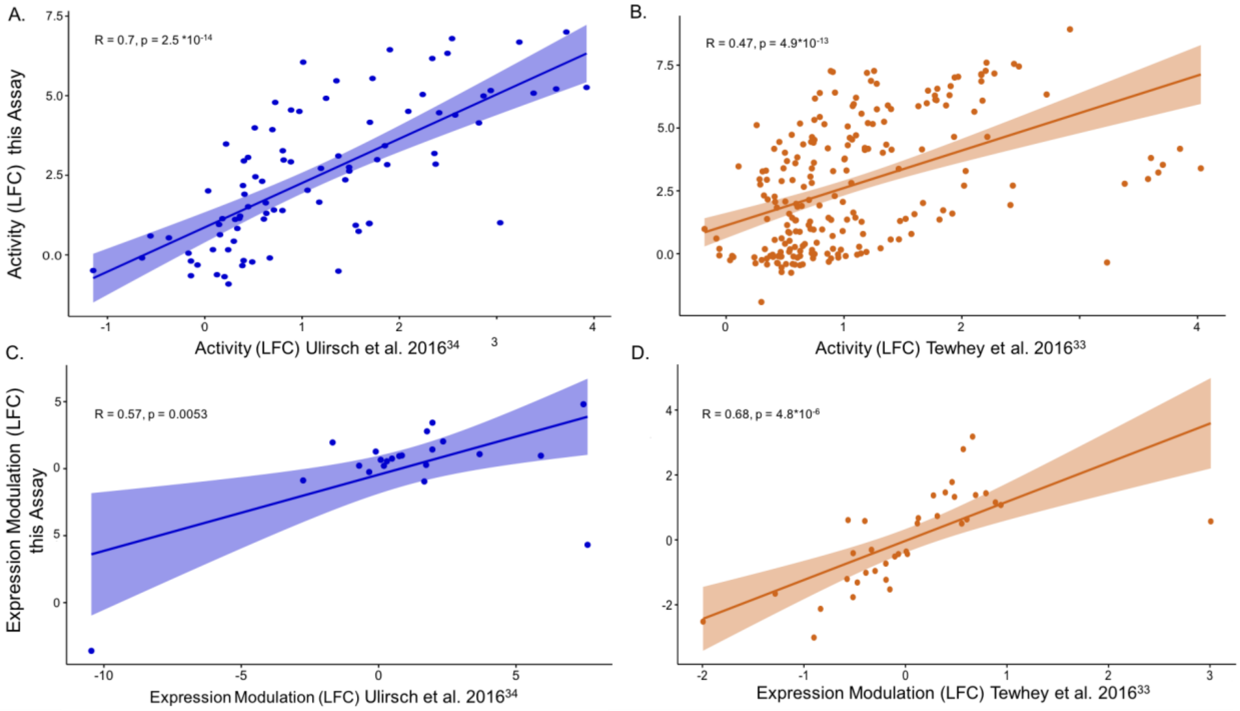

Supplementary Figure 5. Correlation between positive control sequence (A,B) activity and (C,D) expression modulation in their original assay and this MPRA. Positive control oligo sequences derived from two past MPRA experiments (Tewhey et al. 2016; Urlisch et al. 2016). **A** and **B** show the correlation of the activity (LFC) in the original experiment (x--axis), Ulirsch and colleagues (2016)(**A**) and Tewhey and colleagues (2016)(**B**), respectively, with the activity (LFC) in this experiment (y--axis). **C** and **D** show the correlation of the expression modulation (LFC) of sequences in the original (x--axis), Ulirsch and colleagues (2016)(**C**) and Tewhey and colleagues (2016)(**D**), respectively, with the expression modulation (LFC) in this experiment (y-axis). Pearson's R and associated p-value are shown in the upper left corner of each plot.

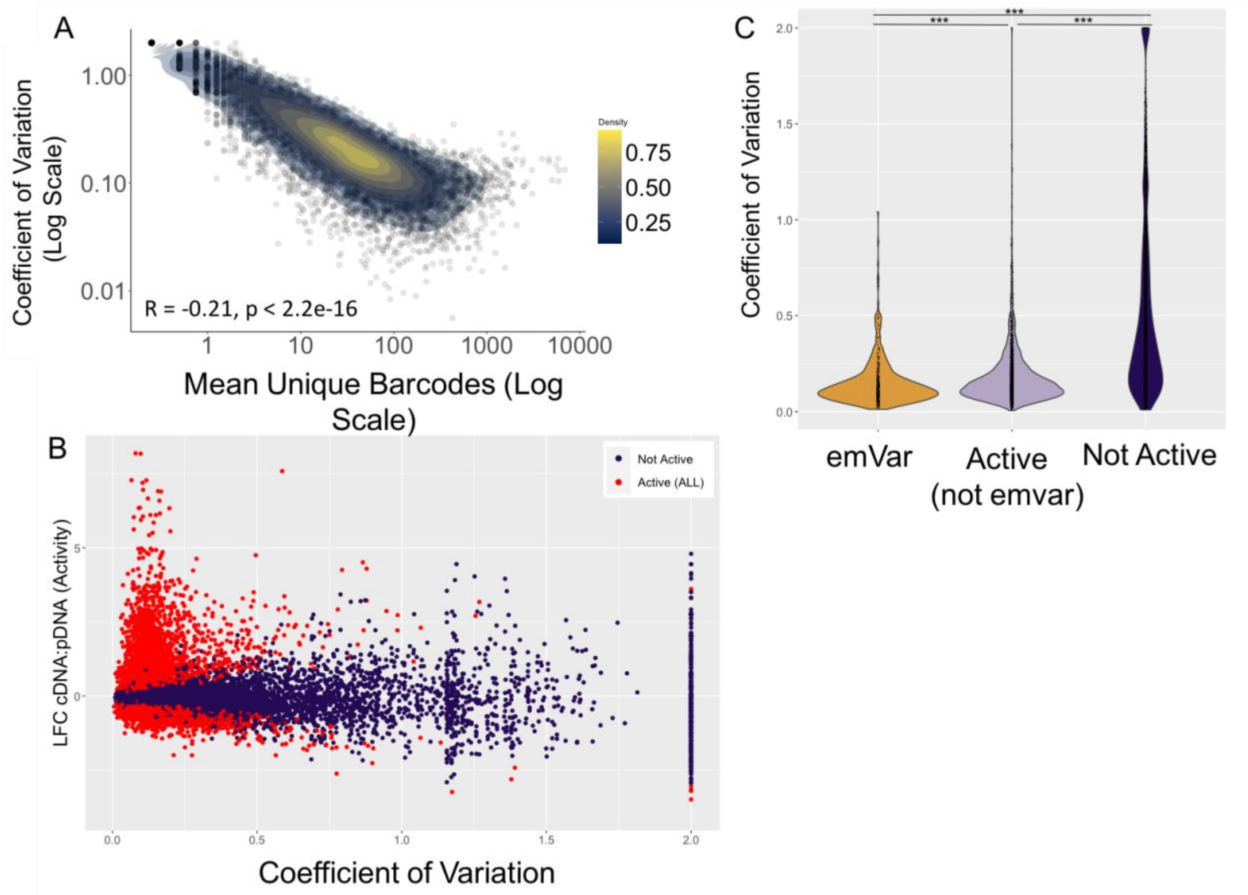

Supplementary Figure 6. Analysis of the relationship between barcode count and coefficient of variation (CV) across experiments. (**A**) Relationship between average unique barcode count across replicates and CV of normalized transcript count (Pearson's r = -0.21, p < 2.2e-16) showing that the majority of oligos have a relative low CV (65% CV < 0.3; 83.5% CV < 0.5%), overall mean = 0.38, and median = 0.21. (**B**) Relationship between the CV and LFC cDNA:pDNA ratio. Points in red were found to be significantly active, while points in blue were not. (**C**) Violin plots showing the relationships of CV across three classes of elements (emVars, active non-emVars, and non-significantly active sequences). In general, we observed significantly lower average CV for emVars (0.15) and active non-emVars (0.18) than non-significantly active sequences (0.53) (t-test emVars vs non-active and active non-emVars vs non-active p-value < 2.2e-16; t-test emVars vs active non-emVars p-value = 5.1e-8).

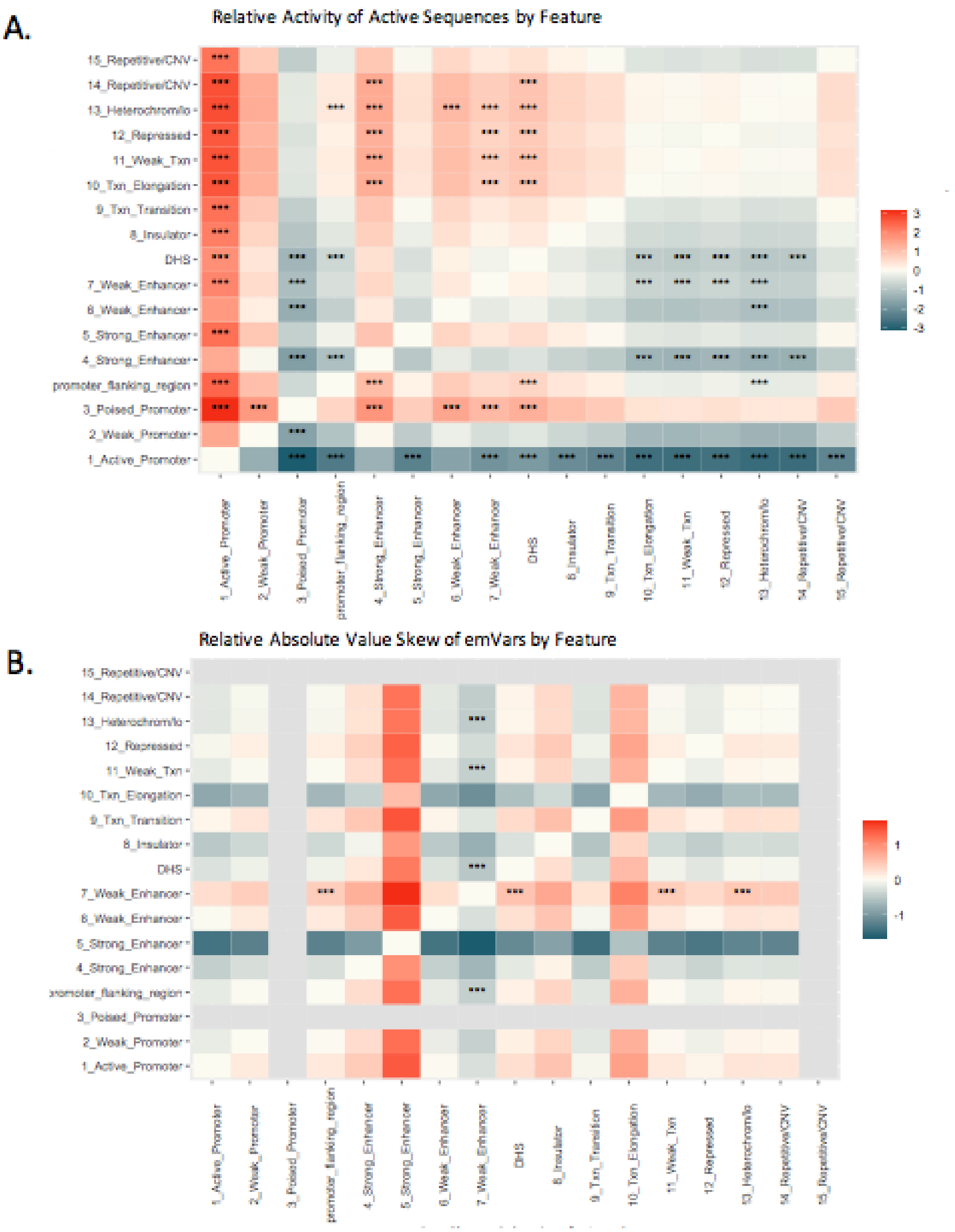

Supplementary Figure 7. Active cis-regulatory elements (CREs) and expression modulating variants (emVars) display prosperities of endogenous K562 biology. (**A**) Relative Average Activity (LFC) of Active Sequences by Feature Enrichment of Active CREs in K562 Genomic Features relative to non-active sequences. (**B**) Relative Absolute Value of Skew of emVars by Feature. Genomic features indicated with a number (i.e., "1_","2_") represent chromatin states in K562 cells as defined by Ernst and Kellis (2017). "All promoter" indicates any of the 1-3 promoter chromatin states. Data for "Promoter

flanking region" derives from Ensembl (Tewhey et al., 2016) and DHS derives from ENCODE (Butler et al., 2014). **A-B** compare the activity (**A**) or absolute expression modulation (**B**) between features. Color gradients represent the difference between the means for each feature. Significance was calculated using a Student's T-Test to compare the means of each features, with stars indicating significance with $p < 0.05$ after correcting for multiple hypothesis testing (Bonferonni method).

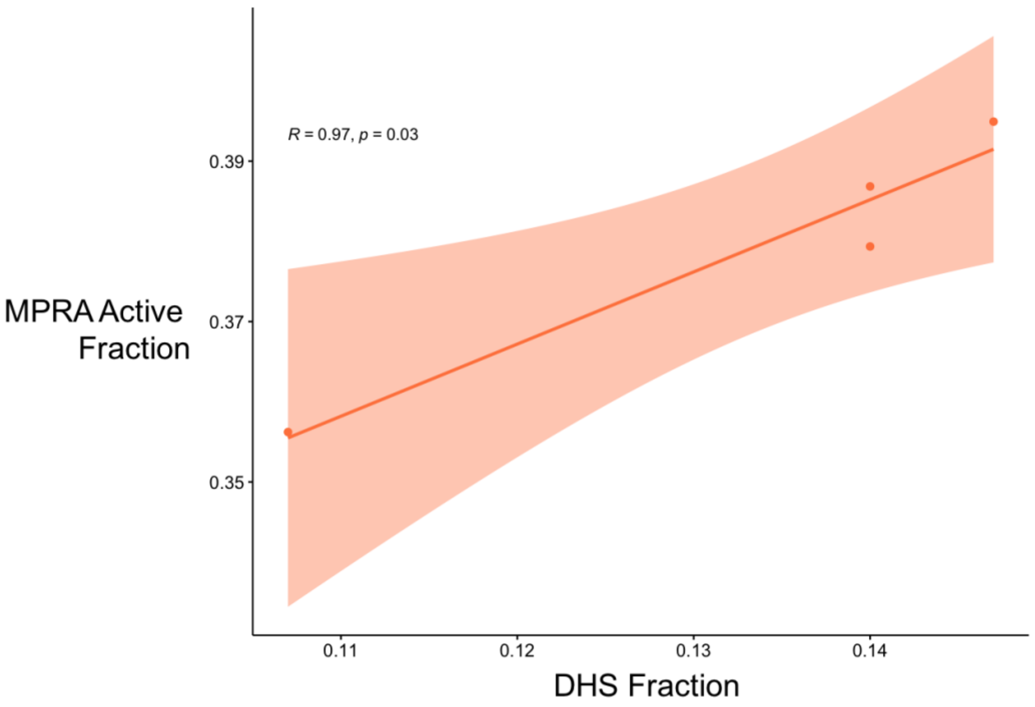

Supplementary Figure 8. Correlation between the proportion of variants within CREs that overlap a K562 DHS fraction and the proportion of variants within active CREs in the MPRA across variant sets. The 4 variant sets shown are the experimental introgressed and non--introgressed sets and the human control sets: human frequency matched only variants, and human frequency and location matched variants. DHS fraction comes from an intersection with ENCODE (Encode Project Consortium 2012) K562 DHS data. Pearson's R and p value are shown in the top left corner of the plot.

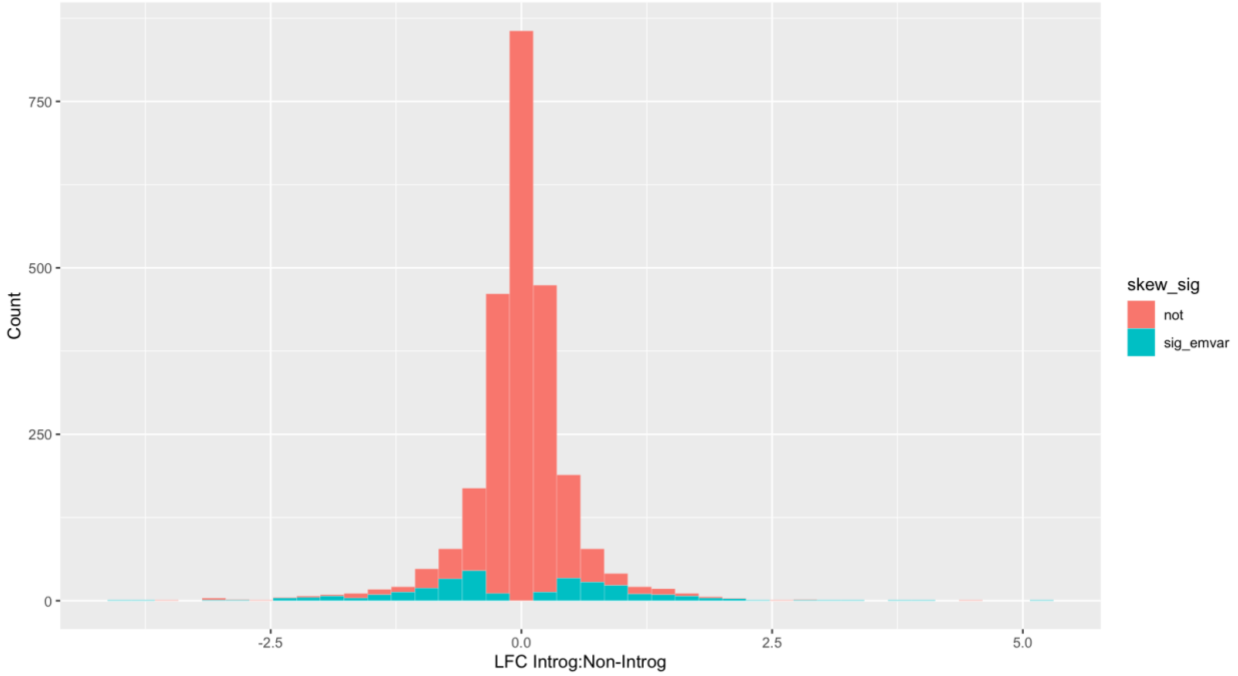

Supplementary Figure 9. Histogram of the LFC introgressed allele: non-introgressed allele for all MPRA variants tested. In this plot, non-significant variants are shown in red and significant emVars (demonstrating significant allelic skew) are shown in blue. emVars showed no overall significant direction of effect based on introgression status and were equally likely to increase or decrease reporter expression in K562 cells (one-sample Wilcoxon Test compared to 0, $p = 0.4$).

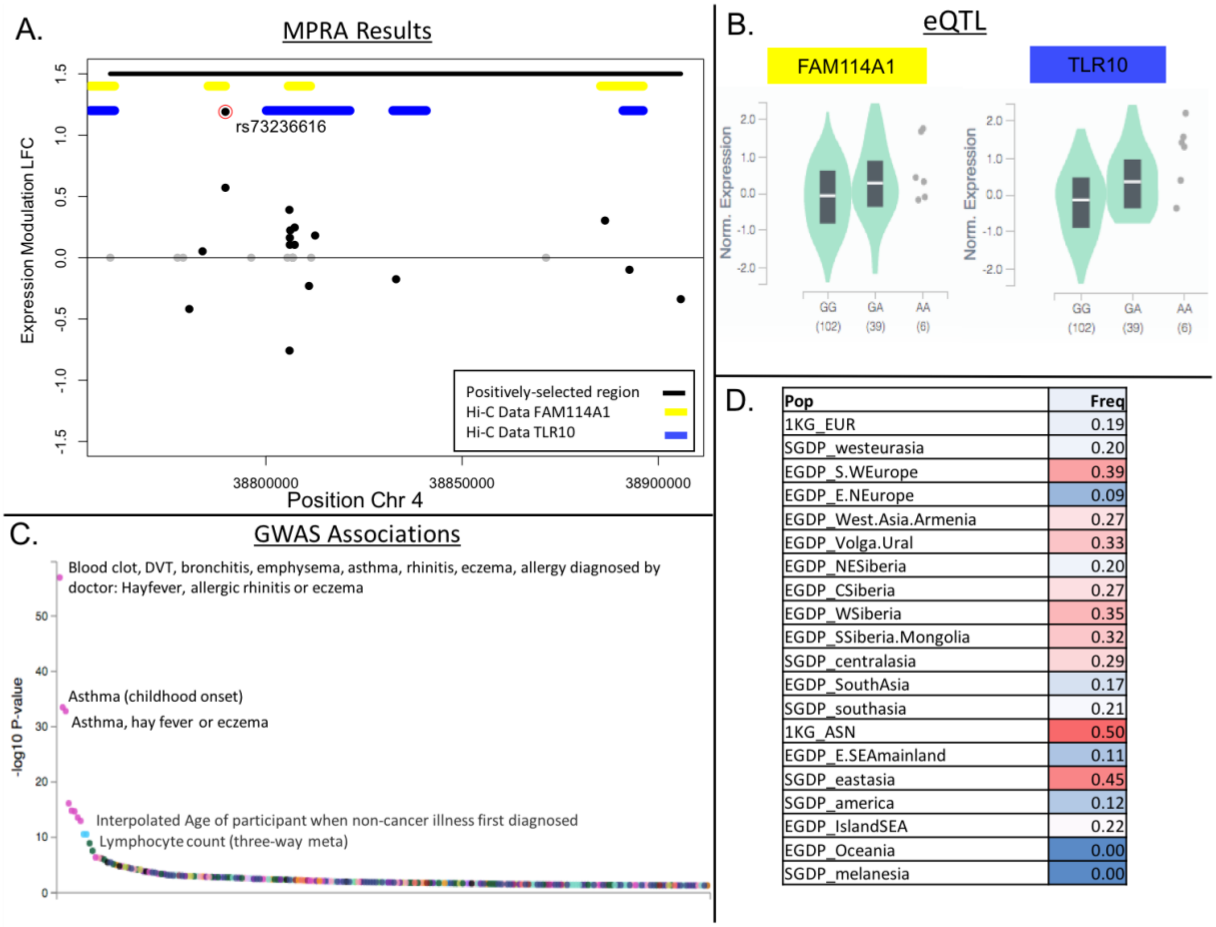

Pop	Freq
1KG_EUR	0.19
SGDP_westeurasia	0.20
EGDP_S.WEurope	0.39
EGDP_E.NEurope	0.09
EGDP_West.Asia.Armenia	0.27
EGDP_Volga.Ural	0.33
EGDP_NESiberia	0.20
EGDP_CSiberia	0.27
EGDP_WSiberia	0.35
EGDP_SSiberia.Mongolia	0.32
SGDP_centralasia	0.29
EGDP_SouthAsia	0.17
SGDP_southasia	0.21
1KG_ASN	0.50
EGDP_E.SEAmainland	0.11
SGDP_eastasia	0.45
SGDP_america	0.12
EGDP_IslandSEA	0.22
EGDP_Oceania	0.00
SGDP_melanesia	0.00

Supplementary Figure 10. MPRA results, eQTL expression information, GWAS associations, and allelic frequencies for test variants across *TLR1-6-10/FAM114A1*. (A) *TLR1-6-10/FAM114A1* locus plot showing the location of variants (dots) tested in MPRA**.** Each point represents a tested variant with grey points indicating variants lying within regions that do not show significant activity in the MPRA. Black points are variants within significantly active CREs. Red circled variants shows significant expression modulation. Black horizontal line depicts positive selection region as detected by Sankararaman et al., 2014; Dannemann et al., 2016; Deschamps et al., 2016; Racimo et al., 2017; Jagoda et al., 2018. Yellow and blue horizontal lines depict Hi-C interactions to *FAM114A1* and *TLR10*, respectively. (**B**) Normalized expression data for *FAM114A1* (left) and *TLR10* (right) for rs73236616:G:A in EBV transformed lymphocytes (data acquired from GTEx). (**C**) GWAS association data from the GWAS Atlas (https://atlas.ctglab.nl/PheWAS). Colored dots indicate different phenotype domains with pink indicating "respiratory" and green indicating "immunological". The full set of GWAS signals for this variant can be found in Table S8. (**D**) Frequency of introgressed allele at rs73236616 in populations analyzed in this study with color scale indicating higher allelic frequencies (pink to red).

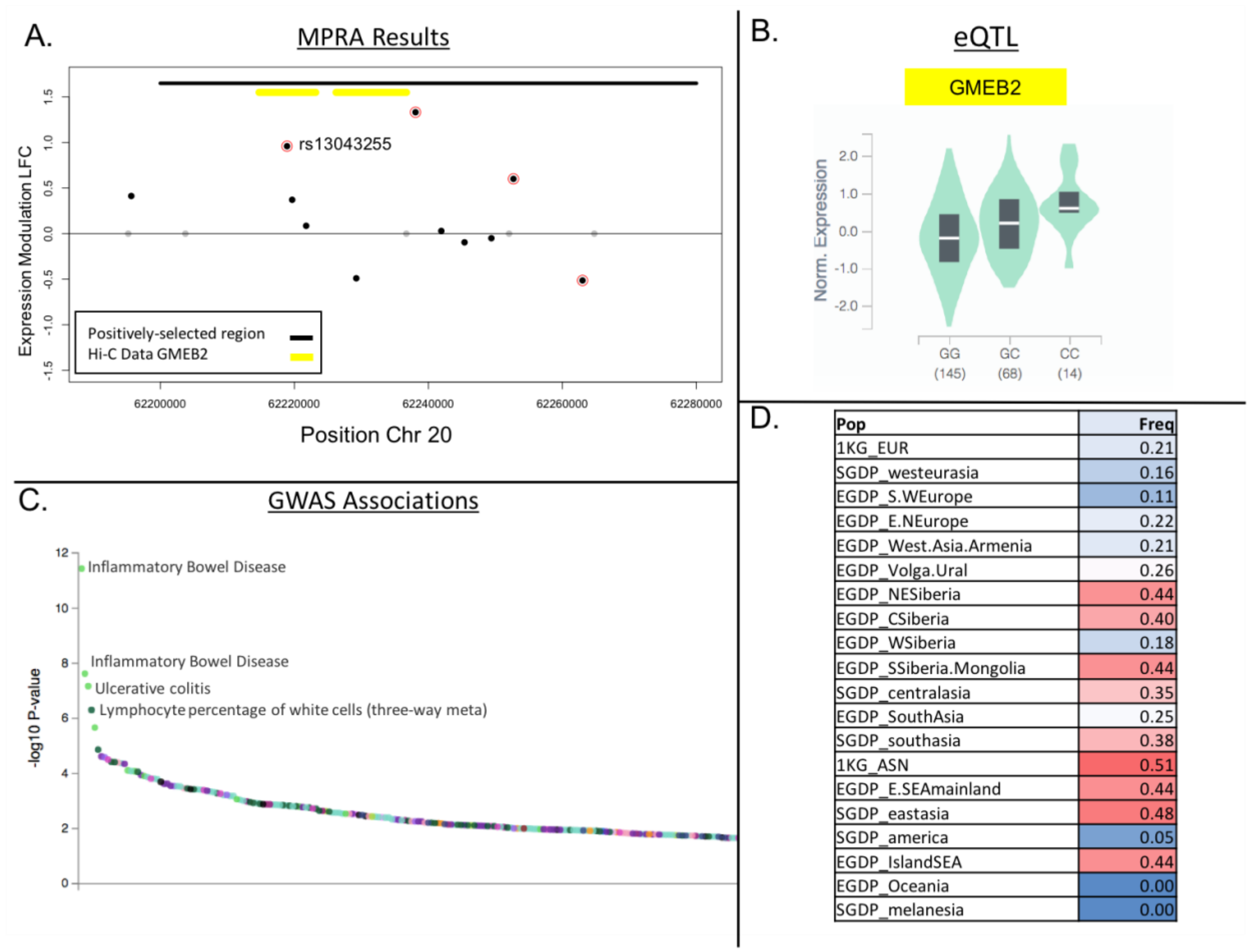

Pop	Freq
1KG_EUR	0.21
SGDP_westeurasia	0.16
EGDP_S.WEurope	0.11
EGDP_E.NEurope	0.22
EGDP_West.Asia.Armenia	0.21
EGDP_Volga.Ural	0.26
EGDP_NESiberia	0.44
EGDP_CSiberia	0.40
EGDP_WSiberia	0.18
EGDP_SSiberia.Mongolia	0.44
SGDP_centralasia	0.35
EGDP_SouthAsia	0.25
SGDP_southasia	0.38
1KG_ASN	0.51
EGDP_E.SEAmainland	0.44
SGDP_eastasia	0.48
SGDP_america	0.05
EGDP_IslandSEA	0.44
EGDP_Oceania	0.00
SGDP_melanesia	0.00

Supplementary Figure 11. MPRA results, eQTL expression information, GWAS associations, and allelic frequencies for eQTLs for *GMEB2*. (A) *GMEB2* locus plot showing the location of variants (dots) tested in MPRA**.** Each point represents a tested variant with grey points indicating variants lying within regions that do not show significant activity in the MPRA. Black points are variants within significantly active CREs. Red circled variants show significant expression modulation. The unlabeled emVars are from left to right rs4809313, rs6089940, rs67114507 (see Table S10). Black horizontal line depicts positive selection region as detected by Sankararaman et al., 2014; Racimo et al., 2017; Jagoda et al., 2018. Yellow horizontal line depicts Hi-C interactions to *GMEB2*. (**B**) Normalized expression data for *GMEB2* for rs13043255:G:C in Spleen (data acquired from GTEx). (**C**) GWAS association data from the GWAS Atlas (https://atlas.ctglab.nl/PheWAS). Colored dots indicate different phenotype domains with light green indicating "gastrointestinal" and dark green indicating "immunological. The full set of GWAS signals for this variant can be found in Table S9. (**D**) Frequency of introgressed allele at rs1304325 in populations analyzed in this study with color scale indicating higher allelic frequencies (pink to red).

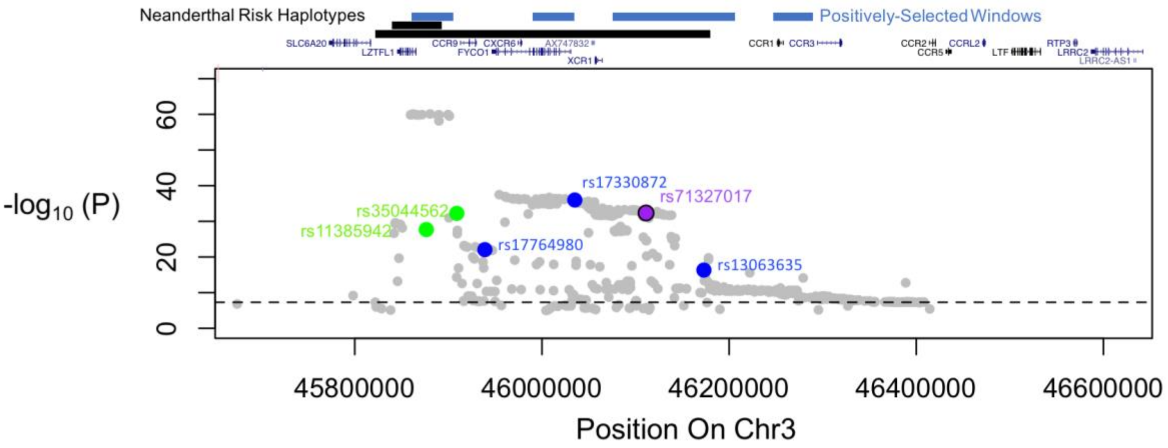

Supplementary Figure 12. The top portion shows the location of the core smaller and longer extended Neanderthal COVID-19 risk haplotypes as defined by Zeberg and Paabo (2020) shown as black rectangles and introgressed and positively selected windows in South Asia as defined by Jagoda and colleagues (2018). Below are the locations of genes within the window adapted from UCSC genome browser (http://genome.ucsc.edu Kent et al. 2002) . The graph below shows variants tested in the GWAS (COVID-19 Host Genetics Initiative) with the –log10(P-value) of their association with COVID-19 severity shown on the y--axis. The index variants described by Zeberg and Paabo (2002) are shown in green. The purple point shows a variant within an active CRE in the MPRA. The blue points are all other MPRA tested variants. Grey points are variants not tested in the MPRA.

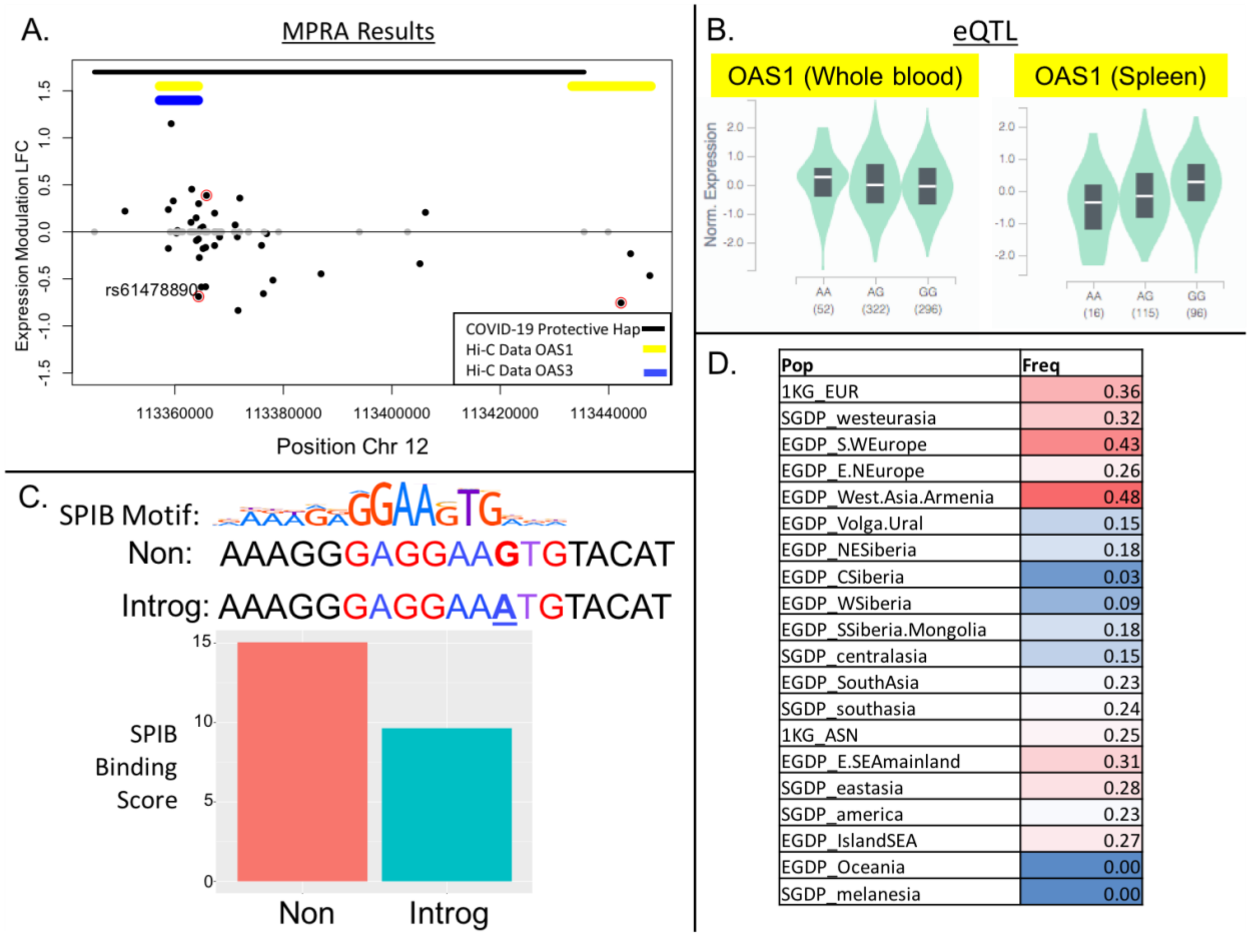

Pop	Freq
1KG_EUR	0.36
SGDP_westeurasia	0.32
EGDP_S.WEurope	0.43
EGDP_E.NEurope	0.26
EGDP_West.Asia.Armenia	0.48
EGDP_Volga.Ural	0.15
EGDP_NESiberia	0.18
EGDP_CSiberia	0.03
EGDP_WSiberia	0.09
EGDP_SSiberia.Mongolia	0.18
SGDP_centralasia	0.15
EGDP_SouthAsia	0.23
SGDP_southasia	0.24
1KG_ASN	0.25
EGDP_E.SEAmainland	0.31
SGDP_eastasia	0.28
SGDP_america	0.23
EGDP_IslandSEA	0.27
EGDP_Oceania	0.00
SGDP_melanesia	0.00

Supplementary Figure 13. MPRA tested variants on Chr12 associated with severe COVID--19 protection and associated eQTL expression information, transcription factor binding analysis, and allelic frequencies. (A) COVID-19 protection locus plot showing the location of variants (dots) tested in MPRA**.** Each point represents a tested variant with grey points indicating variants lying within regions that do not show significant activity in the MPRA. Black points are variants within significantly active CREs. Red circled variants show significant expression modulation; the unlabeled variants from left to right are rs7316586, rs1293749. Black horizontal line depicts COVID-19 protective haplotype as defined by Zeberg and Paabo (2020b). Yellow and blue horizontal lines depict Hi-C interactions to *OAS1* and *OAS3*, respectively. (**B**) Normalized expression data for *OAS1* in whole blood (left) and spleen (right) for rs61478890:G:A (data acquired from GTEx). (**C**) Transcription factor binding analysis for SPIB and its binding affinity to introgressed ("A") and non-introgressed ("G") at rs61478890, showing the reduced (blue bar plot, below) binding affinity for the introgressed allele compared to the non-introgressed allele (red bar plot, below). SPIB motif is shown above the sequences and was acquired from JASPAR (Fornes et al. 2020). Predicted binding scores come from FIMO (Grant et al. 2011). (**D**) Frequency of introgressed

allele at rs61478890 in populations analyzed in this study with color scale indicating higher allelic frequencies (pink to red).
